# Engineering Whole Mammalian Cells for Target‐Cell‐Specific Invasion/Fusion

**DOI:** 10.1002/advs.201700971

**Published:** 2018-05-08

**Authors:** Ryosuke Kojima, Martin Fussenegger

**Affiliations:** ^1^ Department of Biosystems Science and Engineering (D‐BSSE) ETH Zurich Mattenstrasse 26 4058 Basel Switzerland; ^2^ Graduate School of Medicine The University of Tokyo 7‐3‐1 Hongo Bunkyo‐ku Tokyo 113‐0033 Japan; ^3^ Faculty of Science University of Basel Mattenstrasse 26 4058 Basel Switzerland

**Keywords:** biotechnology, cell contacts, cell fusion, cell invasion, synthetic biology

## Abstract

Live mammalian cells are equipped with a synthetic cell invasion system that enables their target‐specific insertion into other live mammalian cells. By conjugating RhoA activator to a transmembrane protein that is segregated from cell–cell interface when specific cell contact occurs, polarization of RhoA activity is synthetically induced inside the cells in response to specific cell contact. This polarization is a sufficient condition for invader cells to selectively penetrate cells expressing a target antigen. Further, when an acid‐responsive fusogenic protein is expressed on invader cells, invader/receiver cell fusion occurs after invasion, and the invader's intracellular contents are released into the recipient's cytosol. It is shown that this system can be used for specific cell ablation. This synthetic‐biology‐inspired cell invasion/fusion system might open the door to using whole mammalian cells for cargo delivery purposes or for ablation of a specific cell type.

In the process of entosis or emperipolesis, a whole living cell invades another living cell.^1^, ^2^ This process is different from endocytosis, in that the cells that are eventually engulfed actively invade the recipient cells. A system that could force cells to invade a specific class of other cells would be an attractive tool for biotechnological use, because, for example, it might allow mammalian cells to be used to deliver a variety of cargoes into target cells. Although synthetic phagocytosis of target cells has been reported,^3^ synthetic cell invasion has not been described. Thus, we set out to develop a synthetic system that would furnish live whole cells with the ability to achieve target‐cell‐specific invasion.

Natural cell invasion is reported to require polarized actin dynamics in the invader cells.^4^, ^5^ More concretely, in an invader cell, RhoA deactivator (e.g., a GTPase‐activating protein for Rho, p190A RhoGAP) accumulates at the cell–cell interface with a receiver cell,^5^ and RhoA activator (e.g., a guanine nucleotide exchange factor (GEF) for Rho, PDZ‐RhoGEF) accumulates at the rear side of the invader cell.^4^ We hypothesized that this polarization of RhoA activity, inside the invader‐cell‐to‐be, is sufficient to cause cell invasion, and thought about how to synthetically induce this polarization in response to specific cell contact. For this purpose, we focused on biophysical movement of a transmembrane protein, CD43_EX_‐45_INT_ (chimeric protein of extracellular and transmembrane domain of CD43 and intracellular domain of CD45). This protein expressed on a mammalian cell bearing an antigen‐recognizing receptor with surface‐displayed single chain antibody (scFv) is reported to be segregated from the cell‐cell interface when the cell binds to some other cells via scFv‐target antigen interaction,^6^, ^7^ while the antigen‐recognizing receptor accumulates at the cell‐cell interface. We hypothesized that we could synthetically cause the polarization of RhoA activity by replacing the intracellular domain of CD43ex‐45int with RhoA in an active form, and the intracellular domain of the antigen‐recognizing receptor with a dominant‐negative form of RhoA (**Figure** **1**a).

**Figure 1:**
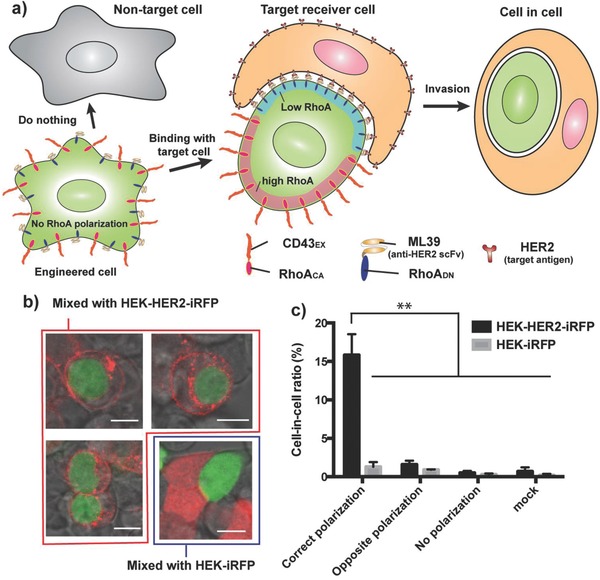
Schematic illustration and characterization of the target‐cell‐specific invasion system. a) Engineered cells are equipped with a chimeric protein consisting of extracellular and transmembrane domains of CD43 and constitutively active RhoA (CD43_EX_‐RhoA_CA_), as well as a chimeric protein of ML39 (HER2 recognition moiety)‐CD28 transmembrane domain‐dominant negative RhoA (ML39‐CD28_TM_‐RhoA_DN_). In the absence of target antigen, RhoA polarization does not occur, so the engineered cells do not invade nontarget cells. Upon binding to HER2 on target cells, the desired polarization of RhoA occurs and the engineered cells invade the target HER2‐positive cells. b) Image of cell‐in‐cell structure (invader cells: yellow; receiver cells: red). Invader cells were transfected with the above‐mentioned invasion components (pRK47: P_hCMV_‐ML39‐CD28_TM_‐RhoA_DN_‐pA, pRK48: P_hCMV_‐CD43_EX_‐RhoA_CA_‐pA) and pEYFP‐C1 (P_hCMV_‐EYFP‐pA), and mixed with HEK‐HER2‐iRFP or HEK‐iRFP cells. After 6 h, the cells were observed under a confocal fluorescence microscope. (Bottom right image is with HEK‐iRFP, and the other three images are with HEK‐HER2‐iRFP cells.) Scale bars indicate 10 µm. c) Quantification of cell‐in‐cell structure. Cells were transfected with the following plasmid together with pEYFP‐C1. Correct polarization: pRK47 and pRK48. Opposite polarization: pRK66 (P_hCMV_‐ML39‐CD28_TM_‐RhoA_CA_‐pA) and pRK67 (P_hCMV_‐CD43_EX_‐RhoA_DN_‐pA). No polarization: pRK64 (P_hCMV_‐RhoA_DN_ (full length)‐pA) and pRK65 (P_hCMV_‐RhoA_CA_ (full length)‐pA). Mock: pcDNA3.1(+). The proportion of the cells that invaded receiver cells among YFP‐positive cells is shown. The ratio of the number of invader cells (including cells that did not take up plasmids) and receiver cells was 1: 1. Error bars represent SEM (*n* = 3; ≈200 cells were observed per experiment, and the average of three independent experiment was calculated). **p < 0.01, two‐tailed Student's *t*‐test.

We first prepared a plasmid encoding CD43, whose intracellular domain was replaced with yellow fluorescent protein (CD43_EX_‐YFP), and a chimeric receptor bearing an extracellular antigen recognition site (model antigen: a well‐known breast cancer marker, human epidermal growth factor receptor 2 (HER2); single‐chain variable fragment (scFv) against HER2: ML39^8^), the transmembrane domain from CD28, and intracellular cyan fluorescent protein (ML39‐CD28_TM_‐CFP). HEK‐293 cells were transfected with these constructs, and were mixed with model target (HEK‐HER2‐iRFP) or nontarget (HEK‐iRFP) cells. Then, the localization of YFP and CFP was observed. As a result, YFP was segregated from the cell–cell interface, while CFP accumulated at the interface only when the cells were mixed with target cells. This result confirms that intracellular part of CD43 can be exchanged without the loss of segregation ability from the cell‐target cell interface that is formed by interaction of target antigen and the newly developed antigen recognition receptor. It was indicated that replacing YFP and CFP directly with active RhoA and dominant‐negative RhoA, respectively, would initiate the desired polarization of RhoA activity (Figure S1a, Supporting Information).

Therefore, we next replaced the intracellular part of CD43 with constitutively active RhoA (RhoA_CA_), in which the CAAX domain is deleted (ΔCAAX) to avoid redundancy of membrane‐localizing domain, and coexpressed it with the antigen recognition receptor whose intracellular part was replaced with dominant‐negative RhoA (RhoA_DN_ (ΔCAAX)) in HEK‐293 cells. In this setting, we expected that the desired polarization of RhoA signaling activity (i.e., higher RhoA activity at the rear side of an invader cell and lower RhoA activity at the interface with the receiver cell) would occur only when the cells become attached to target cells (Figure 1a). Indeed, only when the engineered cells bearing correct RhoA polarization were mixed with target HEK‐HER2‐iRFP cells did we observe cell‐in‐cell structure (Figure 1b,c; for larger‐scale images, see Figure S2 in the Supporting Information). It is noteworthy that the engineered cells invaded only the target cells even in mixed cultures of target and nontarget cells (Figure S3, Supporting Information). These results indicate that it would be possible to use the invader cells as target‐specific delivery vesicles.

Next, we focused on releasing the intracellular contents of invader cells into the cytosol of receiver cells, in order to explore the possibility of delivering various functional molecules into the receiver cells. However, in the process of entosis or emperipolesis, most invader cells are eventually killed by the receiver cells through lysosomal digestion, and some cells even escape from the receiver cells.^1^ We found that cells that were forced to invade target cells by the synthetic cell invasion system met the same fate (Figure S4, Supporting Information). Therefore, to achieve release of the intracellular contents, we expressed a fusogenic protein VSV‐G, which promotes cell fusion under acidic conditions,^9^ on the invader cells, because we thought that the environment of the invader cells after invasion would become acidic due to the initiation of lysosomal digestion of invader cells by the recipient cells (**Figure** **2**a). We found that VSV‐G induced membrane fusion after the invasion, and the intracellular contents of the invader cells were completely released into the cytosol of receiver cells as we had hoped (Figure 2b). We investigated the effect of invader: receiver ratio on invasion/fusion efficacy, and found that invasion/fusion occurs with increasing probability (reaching ≈80%) as the invader cell ratio is increased, indicating that this invasion/fusion system is robust, at least in the current setting (Figure 2c,d and Figure S5, Supporting Information). Also, we confirmed this cell invasion/fusion remained target‐specific even in mixed cultures of target and nontarget cells (Figure S6, Supporting Information).

**Figure 2:**
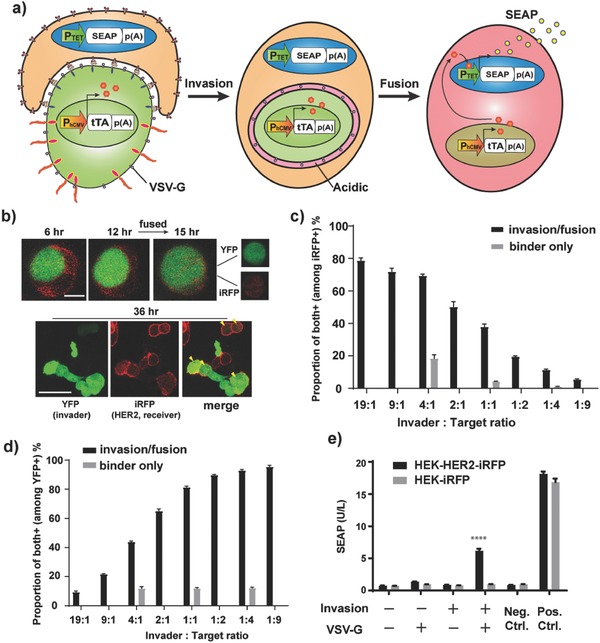
Target‐cell‐specific cell invasion/fusion system. a) Schematic illustration. Invader cells are equipped with the cell invasion components plus VSV‐G, which promotes cell fusion under acidic conditions. Since receiver cells attempt to degrade invader cells by lysosomal digestion, the environment of invader cells becomes acidic after invasion. This promotes fusion of the invaded cells with receiver cells, and the whole intracellular contents of invader cells are released into cytosol of receiver cells. For semiquantitative assay using SEAP reporter, invader cells were transfected with constitutive tTA expression vector, and receiver cells were transfected with tTA‐responsive SEAP expression vector. Upon cell fusion, SEAP expression from the tTA‐responsive promoter is triggered by supply of tTA from the invader cells. With this system, we could evaluate invasion without the need for subjective assessment of cell‐in‐cell structure. b) Images showing cell fusion after invasion. Invader cells were transfected with pEYFP‐C1, pMD2.G (P_hCMV_‐VSV‐G‐pA) together with invasion components (pRK47 and pRK48). The cells were mixed with HEK‐HER2‐iRFP cells and imaging was conducted under a confocal fluorescence microscope. Time above the images indicates time after the start of imaging (imaging was started at 3 h after cell mixing). Yellow arrowheads in “merge” indicate fused cells bearing both YFP and iRFP fluorescence. The scale bar in the upper image indicates 10 µm, and that in lower image indicates 50 µm. c,d) Assessment of the effect of invader: target ratio on invasion/fusion efficacy. The same invader cells as in (b) (for binder‐only cells, only pRK47 and pEYFP‐C1 were transfected) (presorted by FACS using YFP fluorescence) were mixed with HEK‐HER2‐iRFP in various ratios (19:1–1:9. For the binder‐only control, only ratios of 4:1, 1:1, and 1:4 were tested). The cells were seeded in 96 well plates (5 × 10^5^ cells mL^−1^ in total). At 24 h later, the cells were analyzed by FACS. See Figure S5 (Supporting Information) for details of the analysis. c) Proportion of iRFP+/YFP+ cells among iRFP+ cells (reflecting the proportion of receiver cells that were indeed invaded/fused with invader cells). d) Proportion of iRFP+/YFP+ cells among YFP+ cells (reflecting the proportion of invader cells that indeed invaded/fused with target cells). e) Semiquantitative invasion/fusion assay. The receiver cells (HEK‐HER2‐iRFP or HEK‐iRFP) were transfected with pMX9 (P_TET_‐SEAP‐pA), and the invader cells were transfected with pDB24 (P_hCMV_‐tTA‐pA) as well as invasion components (pRK47 and pRK48) and VSV‐G (pMD2.G) (omitted components were replaced with pcDNA3.1(+)). Invader cells were mixed with receiver cells, and SEAP expression was assayed at 24 h after cell mixing. Error bars of all the figures represent SEM (*n* = 3) of three independent experiments. ****p < 0.0001 (against all the other conditions except positive controls), two‐tailed Student's *t*‐test.

The success of this invasion‐triggered cell‐fusion system allowed us to construct a semiquantitative system for evaluation of cell invasion that has higher throughput than observation under a microscope. This evaluation system was designed such that when invasion/fusion occurs, a synthetic transcription factor tetracycline‐dependent transactivator (tTA) transfected into the invader cells and its reporter transfected into the receiver cells encounter each other, leading to expression of the reporter gene secreted alkaline phosphatase (SEAP) (Figure 2a). Using this evaluation system, we confirmed increased reporter gene expression from target receiver cells mixed with engineered cells bearing both invasion and fusion components (Figure 2e; see Figure S7 and Note S1 of the Supporting Information for comparison of suspension and monolayer cultures). This result demonstrates that the invasion system worked well, without the need for subjective judgment of cell‐in‐cell structure, and directly shows that cells equipped with the invasion/fusion system can target‐specifically deliver functional protein into receiver cells. With this system, we also found that the indispensable part of this synthetic invasion system was activation of RhoA at the rear side of the cells rather than repression of RhoA at the cell–cell interface (Figure S8, Supporting Information). (However, as the invasion efficiency did not change so much, we continued to use the antigen recognition receptor with RhoA_DN_ in further studies.) Further, we confirmed that the system also works efficiently with a physiologically more relevant RhoA activator, RhoGEF (catalytic domain of p63RhoGEF^10^), as an effector (Figure S8, Supporting Information). Additionally, the system worked with DARPin^11^ (designed ankyrin repeat proteins; a genetically engineered antibody‐mimetic derived from ankyrin proteins) as an alternative to the antigen recognition moiety, and PDGFR transmembrane domain as a moiety to express the receptor on the cell membrane (Figure S8, Supporting Information and see Figure S1b of the Supporting Information for confirmation of protein segregation with antigen‐recognizing receptor bearing PDGFR transmembrane domain). These results indicate considerable design flexibility of the invasion components. Importantly, this synthetic invasion/fusion system was portable between different invader cell lines (we examined HEK‐293T cells, immobilized human mesenchymal stem cells (hMSC‐TERT), and Hela cells) (Figure S9, Supporting Information), providing further support for the novel finding that RhoA polarization in the invader cell is a sufficient condition for cell invasion to occur.

Next, we assessed the fate of the fused cells after invasion. We expressed a firefly luciferase and tTA‐specific P_TET_ (tetracycline‐responsive promoter)‐driven red‐fluorescent‐protein (dsRed) expression cassette in the receiver cells, and a constitutive tTA expression unit in the invader cells, which allowed sorting of the fused dsRed‐positive cells followed by a proliferation assay utilizing firefly luminescence (**Figure** **3**a). Microscopic analysis and luminescence assay after sorting revealed that most of the fused cells (dsRed+) retained the capacity for protein expression for a while with the two separate nuclei, but did not divide and eventually died within about 10 days (Figure 3b and Figure S10, Supporting Information). The slow increase of the luminescence signal in the dsRed+ population (Figure 3b) was thought to be due to contamination with nonfused cells (fluorescence activated cell sorting (FACS) efficacy did not reach 100%).

**Figure 3:**
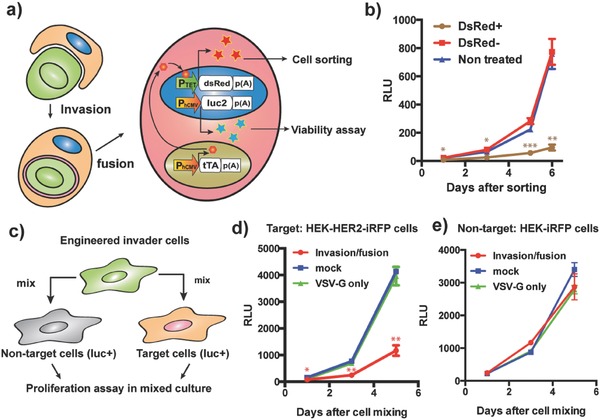
Invasion/fusion system for target‐cell‐specific cell ablation. a) Schematic illustration of the assay for proliferation of fused cells. Receiver HEK‐HER2‐iRFP‐Luc‐ZsGreen cells were transfected with pTREtight‐dsRed (P_TET_‐dsRed‐pA), and invader cells were transfected with pRK47, pRK48, pMD2.G, and pDB24 (P_hCMV_‐tTA‐pA). After cell fusion, tTA drives expression of DsRed, which allows sorting of the fused DsRed+ cells. The dsRed+ cells (as well as equal numbers of dsRed‐ cells and nontreated HEK‐HER2‐iRFP‐Luc‐ZsGreen cells) were seeded on a 96 well plate, and proliferation was traced by firefly luminescence assay. b) Result of the luminescence assay. Some luminescence increase occurred in the dsRed+ population due to contamination with nonfused cells, since we could not completely exclude cell doublets during FACS to capture large fused cells. c) Proliferation assay in the mixed culture condition. Engineered invader cells were mixed with HEK‐HER2‐iRFP‐Luc‐ZsGreen or HEK‐iRFP‐Luc‐ZsGreen cells (invader/receiver = 11/1), and proliferation of receiver cells was traced with firefly luciferase assay. d) Proliferation of HEK‐HER2‐iRFP‐Luc‐ZsGreen cells with invader cells having different components (invasion/fusion: pRK47, pRK48, and pMD2.G). Mock: pcDNA3.1(+), VSV‐G only: pMD2.G. pcDNA3.1(+) was used to replace omitted components. The same number of receiver cells was seeded at Day 0. e) Proliferation of HEK‐iRFP‐Luc‐ZsGreen cells with the same invader cells as in (d). Error bars in all graphs represent SEM (*n* = 3) of three independent experiments. *p < 0.05, **p < 0.01, ***p < 0.001. b) DsRed+ condition was compared to both DsRed‐ and nontreated conditions. d,e) Invasion/fusion condition was compared to both mock and VSV‐G only conditions.

Therefore, we next examined whether the target‐specific cell invasion/fusion system could be used for specific cell ablation. For proof of concept, we prepared model target and nontarget cells stably expressing firefly luciferase (HEK‐HER2‐iRFP‐Luc‐ZsGreen and HEK‐iRFP‐Luc‐ZsGreen, respectively), and mixed them with designed invader cells (Figure 3c). The invader/receiver ratio was set at 11 to increase cell killing efficacy in Figure 3c, and the effect of the invader/receiver ratio on cell killing efficiency is shown in Figure S11 (Supporting Information). (Note that the “invader cells” were not presorted, and so included cells that had not taken up plasmids.) Even without cell sorting after invasion/fusion, we observed clear suppression of the proliferation of only the target cells (Figure 3d,e). This result indicates that designer cells equipped with the target‐specific invasion/fusion system can be used for specific cell ablation.

In summary, we have developed a novel synthetic‐biology‐inspired system that can force mammalian cells to invade specific target cells. We believe it will be possible with this system to use the invader cells as delivery vesicles for various cargo molecules, including proteins and small molecules. This cell‐based delivery system might have advantages over other vesicle‐based delivery systems, because it should be possible to exploit the inherent cell migration properties of certain cell types, such as the tumor tropism of mesenchymal stem cells.^12^ Further, when VSV‐G is coexpressed, the invader cells fuse with the receiver cells after invasion, releasing their whole intracellular contents into the cytosol of the receiver cells. We also showed that this target‐cell‐specific invasion/fusion system is potentially available for specific cell ablation. Because the fused cells remained alive for certain length of time and the protein delivered by invader cells was functional in the fused cells, it might be possible to force the fused cells to exert additional functions that result in a potent bystander effect (for example, expression of a toxic protein to kill surrounding cancer cells),^7^, ^13^ which is not feasible with other cancer ablation methods.

From the viewpoint of future clinical applications, it will be necessary to create invader cells stably equipped with invasion/fusion components. In this context, we confirmed that expression of the invasion components did not kill the invader cells on the time scale of transient transfection (Figure S12, Supporting Information). In addition, cells stably expressing RhoA have been reported,^14^ so it could be possible to construct stable invader cells. However, stable expression of VSV‐G is reported to be toxic for cells,^15^ so further work will be needed to establish that the present proof‐of‐concept study can be translated into practical applications. A promising strategy could be to engineer the invasion/fusion components under the control of specific‐cell‐contact‐sensing transgene expression devices.^7^, ^16^ If we wish to use the invasion/fusion system for pure delivery purposes, the fact that the fused cells did not proliferate normally is problematic. However, it may be worth trying to use enucleated cells as invader cells to overcome this issue (this system would work in enucleated cells, since it does not require transcription/translation steps), because it is possible that the presence of multiple nuclei in one cell, an unusual situation for the cell, may be the reason why proliferation stopped. Further study of these issues, as well as investigation of the generalizability of the target and the in vivo behavior of the invader cells will be necessary for future applications.

Nevertheless, we believe that this first‐in‐class synthetic target‐cell‐specific invasion/fusion system is biologically very interesting, and might open the door to using engineered mammalian cells as “Trojan horses” for killing or delivering various molecules to specific target cells.

## Conflict of Interest

The authors declare no conflict of interest.

## Supporting information

Supplementary
