# Supplementary material for: Engineering Whole Mammalian Cells for Target‐Cell‐Specific Invasion/Fusion
Source: Adv Sci (Weinh). 2018 May 8;5(7):1700971. doi: 10.1002/advs.201700971 (PMC6051388; doi:10.1002/advs.201700971)
Supplement: Supplementary file 1 — Supplementary [file ADVS-5-1700971-s001.pdf]

Copyright WILEY-VCH Verlag GmbH & Co. KGaA, 69469 Weinheim, Germany, 2018.

ADVANCED SCIENCE
Open Access

Supporting Information

for *Adv. Sci.,* DOI: 10.1002/advs.201700971

Engineering Whole Mammalian Cells for Target-Cell-Specific Invasion/Fusion

*Ryosuke Kojima and Martin Fussenegger**

Copyright WILEY-VCH Verlag GmbH & Co. KGaA, 69469 Weinheim, Germany, 2017.

Supporting Information

Engineering Whole Mammalian Cells for Target-Cell-Specific Invasion/Fusion

*Ryosuke Kojima, Martin Fussenegger**

Inventory

Methods……………………………………………………………………………………………...2

Supplementary figures and tables

Figure S1. Confirmation of protein translocation upon specific cell contact………………………..8

Figure S2. Observation of cell invasion (larger-scale images)…………………………………….....9

Figure S3. Target-cell-specific invasion in the mixed culture of target cells and non-target cells….10

Figure S4. Cell fate observed after cell invasion…………………………………………………11

Figure S5. Assessment of the effect of invader : target ratio on invasion/fusion efficacy…………..12

Figure S6. Target-specific invasion/fusion in the mixed culture of target and non-target cells……..14

Figure S7. Comparison of the result of tTA delivery-based invasion/fusion assay between a cell-adhesion condition and a cell-suspension condition……………….………………...……15

Figure S8. System performance using different antigen binders, transmembrane domains, and an effector………………………………………………………………………………16

Figure S9. Portability of the cell invasion/fusion system to different cell lines……………………..17

Figure S10. Fate of fused cells…………………………………………………………………18

Figure S11. Effect of invader/receiver ratio on the cell killing effect……………………………….19

Figure S12. Assessment of the viability of invader cells……………………………………………20

Note S1. Note for cell-suspension effect…………………………………………………………….21

Table S1. Description of the plasmids used in this study……………………………………………22

Table S2. Table of the oligonucleotides used for cloning……………………………………………23

Supporting references……………………………………………………………………….24

Methods

Cell culture and transfection

HEK-293T cells (DSMZ: ACC-635), HEK-293 cells (DSMZ: ACC-305) and their stable transfected cell lines (HEK-293-HER2-iRFP (called HEK-HER2-iRFP) cells, HEK-293-iRFP (called HEK-iRFP) cells, HEK-293-HER2-iRFP-luc-ZsGreen (called HEK-HER2-iRFP-Luc-ZsGreen), HEK-293-iRFP-luc-ZsGreen (called HEK-iRFP-Luc-ZsGreen), and human mesenchymal stem cells transgenic for the catalytic subunit of human telomerase (hMSC-TERT)[1] were cultivated in DMEM (Invitrogen) supplemented with 10% (v/v) fetal bovine serum (FBS, Sigma-Aldrich) and 1% (v/v) penicillin/streptomycin solution (Sigma-Aldrich) at 37 °C in a humidified atmosphere containing 5% CO_2. 400 µg/ml of G418 (Sigma) was also added to cultures of HEK-HER2-iRFP, HEK-iRFP, HEK-HER2-iRFP-Luc-ZsGreen, and HEK-iRFP-Luc-ZsGreen cells. For serial passage of these cells, 0.05% or 0.25 % Trypsin-EDTA (Gibco) was used. For transfection, $2.5x10^5$ cells/ml of cells (counted with a Casy® TTC Cell Counter (OMNI Life Science) or a Countess® Automated Cell Counter (Thermo Fisher Scientific)) were seeded on a 24-well plate (Thermo Fischer Scientific) (500 µL/well) 24 hours before transfection. DNA-polyethyleneimine (PEI) mixture (50 µL) was produced by incubating 2.5 µL PEI (PEI, 20000 MW, Polysciences; stock solution 1 mg/ml in dH_2O) with 500 ng of total DNA, followed by vortexing for 1 s and incubation at r.t. for 15 min. (When necessary, transfection mix and cells used for transfection were scaled appropriately for 12-well plates, 6-well plates, or 10 cm culture dishes; the amounts of DNA and reagent were changed accordingly.) Before the transfection, cell culture medium was replaced with fresh medium containing 10% FBS. The cells were incubated with the transfection mixture for 8-16 hours. Subsequently, the medium was replaced again with fresh, pre-warmed medium for expression of the gene of interest. Establishment of HEK-HER2-iRFP, HEK-iRFP, HEK-HER2-iRFP-Luc-ZsGreen, and HEK-iRFP-Luc-ZsGreen cells has been reported elsewhere[2].

SEAP assay

The supernatant obtained from the transfected cells was incubated at 65°C for 30 min to inactivate endogenous alkaline phosphatase, and 80 µL of the heat-inactivated medium was placed in wells of a transparent 96-well plate. Then, 100 µL of 2 × SEAP buffer (20 mM homoarginine, 1 mM $MgCl_2$, 21% (v/v) diethanolamine, pH 9.8) and 20 µL of p-nitrophenyl phosphate (pNPP) solution (120 mM) were added. The time course of absorbance at 405 nm was measured at 37 °C with an EnVision 2104 Multilabel Reader. SEAP production in U/L was quantified from the slope of the time-dependent increase

in absorbance by using an equation established with appropriate standards. All measurements were made in the region where the relationship was linear in order to avoid saturation effects.

Firefly luciferase viability assay

D-Luciferin (final 500 µM) was added to wells containing cells expressing firefly luciferase (in 96-well plates), and the plate was incubated for 10 min. The luminescence was measured with a Tecan M200 Infinite Pro plate reader. The ratio of luminescence intensity to the control was used as relative cell viability.

Poly-HEMA coating of the dishes

A solution of 6 mg/ml poly(2-hydroxyethyl methacrylate) (poly HEMA, Santa Cruz Biotechnology) in 95 % ethanol was prepared and used to coat the plates or slides (100 µL for a 24-well plate, 50 µL for an Ibidi µ-Slide 8 well), which were then dried under a cell culture hood overnight and used for experiments.

Observation of invasion and invasion/fusion

HEK-293 or HEK-293T cells were transfected with invasion or invasion/fusion components. These cells were mixed with HEK-HER2-iRFP and/or HEK-iRFP cells, and observed with a Nikon Confocal A1 or Leica SP8 microscope (usually equipped with a x63 oil lens).

Figure-specific protocols are as follows.

Fig. 1b: HEK-293 cells were transfected with pRK47, pRK48, and pEYFP-C1 (167 ng each per well of a 24-well plate). (Hereinafter, plasmid amounts are given as those per well of a 24-well plate unless otherwise specified; if necessary, the cell culture and transfection protocols were appropriately scaled to a 12-well plate, 6-well plate, or 10 cm dish.) At 24 hr after transfection, cells were trypsinized, spun down, and suspended in 40 µL of DMEM with 10 % FBS and P/S (hereinafter, always with 10% FBS and P/S unless otherwise specified). This cell suspension was divided into two aliquots (20 µL x 2), and mixed with another cell suspension (20 µL) containing approximately $4x10^5$ HEK-HER2-iRFP or HEK-iRFP cells. The cell suspension was incubated at 37 °C in a 1.5 mL tube. Then, 250 µL of DMEM was added, and the cells were seeded on an Ibidi µ-Slide 8 well. At 4 hrs after seeding, the cells were observed with a Nikon Confocal A1 microscope.

Fig. 2b, S2: HEK-293 cells were transfected with pRK47, pRK48, pEYFP-C1, and pMD2.G (125 ng/each per well of a 24-well plate). At 24 hr after transfection, cells were detached by Cell Dissociation Buffer (Thermo Fischer Scientific), spun down, and suspended in 60 µL of DMEM. Then, 20 µL of the cell suspension was mixed with 20 µL of another suspension containing approximately $2.5x10^5$ HEK-HER2-iRFP or HEK-iRFP cells. The mixture was incubated at 37 °C in a 1.5 mL tube. Then, 250 µL of DMEM containing 0.4% low-melting agarose (Sigma, A4018) were added, and the cells were seeded on an Ibidi µ-Slide 8 well. The slide was left at room temperature until the medium solidified, and

then mineral oil was applied on the top of the solidified medium to prevent it from drying out. This slide was observed with a Nikon Confocal A1 microscope.

Fig.S2. The same protocol as described for Fig. 1b was used. Larger-scale images are shown.

Fig.S3. The protocol was basically the same as for Fig. 1b. The receiver cells were a mixture of HEK-HER2-iRFP and HEK-iRFP cells.

Fig.S4: (a) The same protocol as described for Fig.1b was used, but the cells were observed for a longer time. (b) Cells were prepared according to the same protocol as for Fig.1b, then DMEM containing 0.4% low-melting agarose was added to the cell mixture, and the cells were seeded on an Ibidi µ-Slide 8 well coated with PolyHEMA. The slide was left at room temperature until the medium solidified, and observed with a Nikon Confocal A1 microscope.

Fig. S6. The protocol was similar to that described for Fig. 1b. Differences were as follows. HEK-293T cells transfected with pRK47, pRK48, pRK85 (P_{SV40}-mCherry-pA), and pMD2.G (125 ng each per well of a 24-well plate) were used. The invader cells were mixed with a mixture of HEK-HER2-iRFP-Luc-ZsGreen and HEK-iRFP cells. The mixed cells were seeded on an Ibidi µ-Slide 8 well. At 4 hrs after seeding, the cells were observed with a Leica SP8 microscope. The tile scan mode was used to obtain images, which is why the DIC image has a grid-like pattern.

Quantification of invasion using microscopy data.

HEK-293 cells were transfected as described in the legend of Fig.1c (167 ng of each plasmid). The subsequent procedure was as described for Fig. S2 under each condition. Approximately 200 cells were observed for each condition per experiment, and the cell-in-cell ratio was calculated. For this purpose, cells completely surrounded by single receiver cells exhibiting iRFP fluorescence were treated as cells forming cell-in-cell structures (for example, when 30 invader cells formed cell-in-cell structures, the cell-in-cell ratio was calculated to be 15 %). The experiment was independently conducted three times under each condition, and average ratio of the 3 repeated experiments is shown in the graph. Only 1 focal plane was observed per experiment, so it is possible that in some cases the invader cells were not completely enclosed inside the receiver cells. However, the results were consistent with the invasion-fusion-based reporter assay, suggesting that the microscope observations were valid (see also the legend of Fig.1c)

Evaluation of invasion/fusion by reporter gene assay

Invader cells were transfected with invasion components, pMD2.G ($P_{EF1\alpha}$-VSV-G-pA) and pDB24 (P_{hCMV}-tTA-pA), while receiver cells were transfected with pMX9 (P_{TET}-SEAP-pA), and the two were mixed. After 24 hours, SEAP activity in the cell culture supernatant was measured. Figure-specific protocols are as follows.

Fig.2e: HEK-293 cells were transfected with pRK47, pRK48, pDB24, and pMD2.G (125 ng each; for the

negative (–) condition, pcDNA3.1(+) was used as a filler). In parallel, receiver HEK-HER2-iRFP and HEK-iRFP cells were transfected with pMX9 (250 ng pcDNA3.1(+) was used as filler, and for positive controls, 62.5 ng of pDB24 was also co-transfected). At 24 hr after transfection, the cells were detached with Cell Dissociation Buffer, spun down, and suspended in 60 µL of DMEM. Then, 20 µL of invader cell suspension and 20 µL of receiver cell suspension was mixed, and incubated at 37 °C in a 1.5 mL tube. Next, 500 µL of DMEM was added, and the cells were seeded on a 24-well plate. After 24 hr, SEAP activity in the cell-culture supernatant was assayed.

Fig.S7. The protocol until cell mixing was the same as described for Fig.2c. The mixed cells were seeded on either a normal 24-well plate or a poly-HEMA-coated 24-well plate, and SEAP activity in the supernatant was measured at 24 hours after seeding the cells.

Fig.S8. Invader HEK-293 cells were transfected as described in the figure legend, using 125 ng of each binder, 125 ng of effector (pRK48 or pRK253), 125 ng of pDB24, and 125 ng of pMD2.G. For the negative (–) condition, pEYFP-C1 was used as a filler. Then, the same assay as described for Fig.2c was conducted.

Fig.S9. The same procedure as described in Fig.2c was conducted with HEK-293, HEK-293T, hMSC-TERT, Hela, and CHO-K1 cells as potential invader cells.

Evaluation of cell viability of the receiver cells

Figure-specific protocols are given below.

Fig.3b: HEK-293 cells were transfected with pRK47, pRK48, pDB24, and pMD2.G (500 ng each/well of a 6-well plate; 2 wells were prepared for each condition). In parallel, HEK-HER2-iRFP cells stably expressing Luc-Zsgreen were transfected with pTREtight-dsRed (1000 ng/well of a 6-well plate; 2 wells were prepared, and pcDNA3.1(+) was used as a filler). At 4 hr after transfection, the cells were detached with Cell Dissociation Buffer, spun down, and suspended in DMEM (invader & receiver: 480 µL each) The cell suspensions were mixed and incubated at 37 °C in a 1.5 mL tube. Then, 6 mL of DMEM was added, and the cells were seeded in 3 wells of a 6-well plate. At 24 hrs after seeding, cell sorting to analyse iRFP+, ZsGreen+, dsRed± was conducted with a BD Influx cell sorter. 6500 cells of dsRed+ population, dsRed- population, or non-treated HEK-HER2-iRFP-Luc-ZsGreen were suspended in 3 mL of DMEM, and 110 µL of each cell suspension was seeded on a 96-well plate. Cell viability assay was conducted by firefly luminescence assay at the indicated time points.

Fig.3d,e,S11: HEK-293 cells were transfected with pRK47, pRK48, pDB24, and pMD2.G (500 ng each/well in a 6-well plate for the invasion-fusion condition; for the mock condition, 2000 ng of pcDNA2.1(+) was transfected; for the VSV-G-only condition, 500 ng of VSV-G and 1500 ng of pcDNA3.1(+) were transfected). At 24 hr after transfection, the cells were detached with Cell Dissociation Buffer, spun down, and suspended in 300 µL of DMEM. At the same time, equal numbers of HEK-HER2-iRFP-Luc-ZsGreen and HEK-iRFP-Luc-ZsGreen cells were suspended in 300 µL of DMEM.

Then, 110 µL of invader cell suspension and 10 µL of receiver cell suspension were mixed (in Fig.S8, the following amounts were used; 60 µL each for 1:1 mixture, and 100 µL of invader cells and 20 µL of receiver cells for 5:1 mixture). The mixture was incubated at 37 °C in a 1.5 mL tube, 500 µL of DMEM was added, and the cells were seeded on a 24-well plate. After 1 day, the cells were trypsinized, spun down, and suspended in DMEM to give 4-fold- or 20-fold-diluted cell culture (compared to the 24-well plate culture after cell mixing). This cell suspension was seeded on a 96-well plate (110 µL/well), and cell viability was determined by firefly luminescence assay. (Four-fold dilution was used for measurement at 1 or 3 days after seeding, and 20-fold dilution was used for measurement at 5 days after seeding; luminescence intensity at 5 days after mixing was multiplied by 4 to compare it with the results at 1 and 3 days.)

Confirmation of protein segregation by fluorescence imaging

HEK-293T cells were transfected with the plasmids indicated in the figure legend in a well of 24-well plate (250 ng/each plasmid). At 24 hrs after transfection, cells were detached with TrypLE express, spun down, and suspended in 60-100 µL of DMEM. 20 µL of this cell suspension was mixed with the same number of HEK-HER2-iRFP or HEK-iRFP cells, and the mixture was incubated at 37 °C in a 1.5 mL tube. Then, 500 µL of DMEM was added, and 250 µL of the suspension was seeded on an Ibidi µ-Slide 8 well. After 3-5 hrs, the cells were observed with Nikon Confocal A1 (Fig. S1a) or Leica SP8 (Fig. S1b) microscope.

Assessment of the effect of invader : target ratio on invasion/fusion efficacy by FACS.

HEK-293T cells were transfected with pRK47, pRK48, pEYFP-C1, and pMD2.G (3000 ng of pRK47, 48, and pMD2.G as well as 1000 ng of pEYFP-C1) for the “invasion/fusion” group. HEK-293T cells were transfected with pRK47, pEYFP-C1 and pcDNA3.1(+) (3000 ng of pRK47, 1000 ng of pEYFP-C1, and 6000 ng of pcDNA3.1(+)) for the “binder-only” condition. At 24 hours after transfection, the invader (or binder control) cells were sorted with a FACS AriaIIIu (BD). For the subsequent protocol, see the legend of Fig. S5. For observation of the YFP+/iRFP+ population, the mixed cells were sorted with a FACS AriaIIIu at 24 hours after mixing, seeded on an Ibidi µ-Slide 8 well, and observed with a Leica SP8 microscope.

Observation of the fate of fused cells.

See the following figure-specific protocols.

Fig.S10a: Invader HEK-293 cells (per well of a 6-well plate) were transfected with 500 ng of pRK48, 500 ng of pRK47, 500 ng of pDB24, 250 ng of H2b-Citrine, and 250 ng of Lyn-YFP. The receiver HEK-HER2-iRFP cells were transfected with 1500 ng of pTREtight-dsRed (pcDNA3.1(+) as a filler). At 24 hrs after transfection, the nuclei of receiver cells were stained with NlucBlue Live (ThermoFischer

Scientific). Then, the invader and receiver cells were trypsinized, spun down, and suspended in 240 µL of DMEM. The invader and receiver cell suspensions were mixed and the mixture was incubated at 37 °C in a 1.5 mL tube. Then, 2 mL of DMEM was added, and the cells were seeded in a 6-well plate (poly-HEMA coated). After 24 hours, the YFP+ iRFP+ dsRed+ population was sorted with a BD Influx cell sorter. The cells seeded on an Ibidi µ-Slide 8 well, and observed with a Nikon Confocal A1 microscope.

Fig.S10b: Invader HEK-293T cells were transfected with 2300 ng each of pRK48, pRK47, pDB24, and pMD2.G, as well as 800 ng of pEYFP-C1 (in a 10 cm dish). The receiver HEK-HER2-iRFP cells were transfected with 3000 ng of pTRE-tight-dsRed and 7000 ng of pcDNA3.1(+). At 1 day after transfection, the cells were trypsinized, mixed with the same number of HEK-HER2-iRFP cells, and seeded on two 10 cm dishes (Day 0). After 2 days, YFP+/iRFP+/DsRed+ cells were sorted with a FACS Aria IIIu. For the subsequent protocol, see the corresponding figure legend. Calcein-AM was purchased from Dojindo (C326), and EthD-1 was purchased from ThermoFisher Scientific (E1169).

Observation of the fate of invader cells.

Invader HEK-293T cells were transfected with 600 ng each of pRK47 and pRK48, as well as 800 ng of pRK22 (P_{hCMV}-iRFP-pA). At 24 hours after transfection, iRFP-positive cells were sorted with a FACS AriaIIIu. For the subsequent protocol, see the corresponding figure legend (Fig. S11)

Supplementary Figures

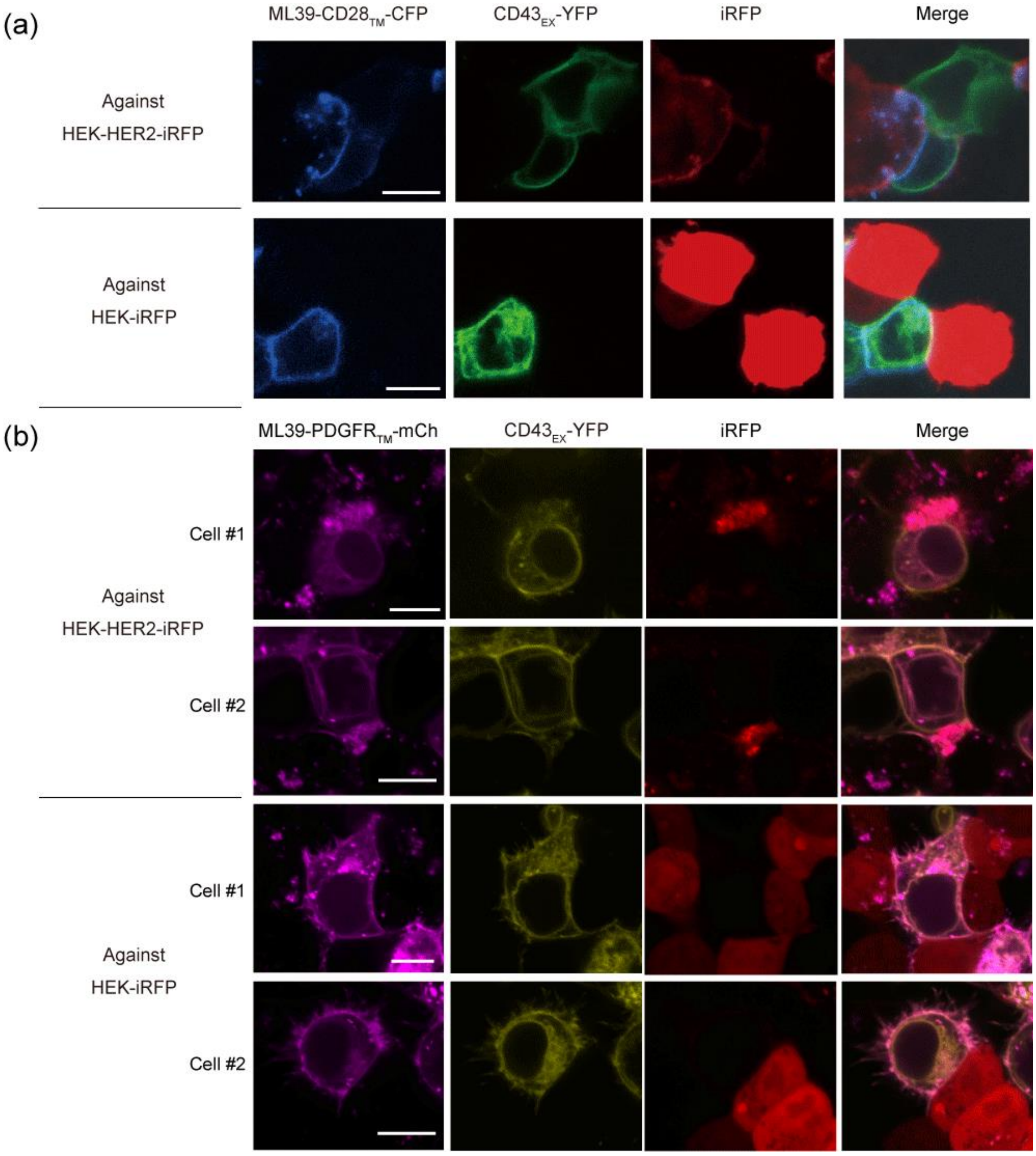

Figure S1. Confirmation of protein translocation upon specific cell contact. Sensor HEK-293T cells were transfected with the indicated plasmids, and mixed with HEK-HER2-iRFP or HEK-iRFP cells. The mixed cells were observed with a confocal microscope. (a) Sensor cells were transfected with pRK14 (P_{hCMV}-CD43$_{EX}$-YFP-pA) and pRK34 (P_{hCMV}-ML39-CD28$_{TM}$-CFP-pA) (the fluorescence of two HEK-iRFP cells is saturated, but this was necessary to show another HEK-iRFP cell having weaker fluorescence adjacent to the sensor cell). (b) Sensor cells were transfected with pRK14 (P_{hCMV}-CD43$_{EX}$-YFP-pA) and pRK256 (P_{hCMV}-ML39-PDGFR$_{TM}$-mCherry-pA). As shown in both (a) and (b), only when the sensor cells contact with target cells was CD43$_{EX}$-YFP segregated from the cell-cell interface, while antigen-recognizing receptor accumulated there. Scale bars indicate 10 μm.

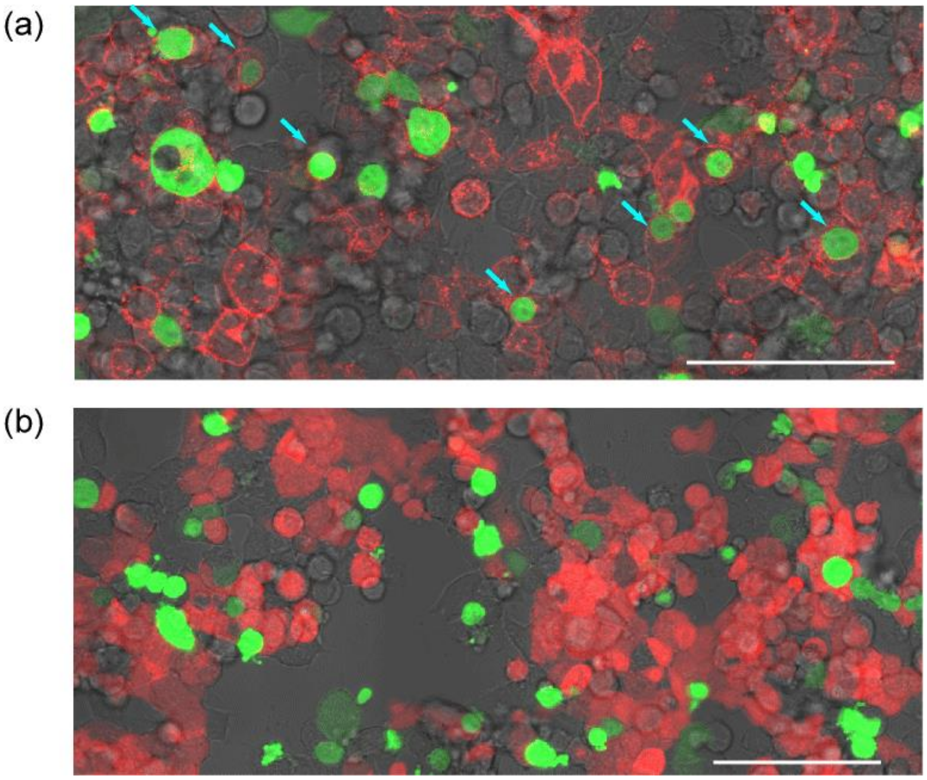

Figure.S2 Observation of cell invasion (larger-scale images). HEK-293 cells were transfected with pRK48 (P_{hCMV}-CD43$_{EX}$-RhoA$_{CA}$-pA), pRK47 (P_{hCMV}-ML39-CD28$_{TM}$-RhoA$_{DN}$-pA), and pEYFP-C1 (P_{hCMV}-EYFP-pA), and mixed with either HEK-HER2-iRFP or HEK-iRFP cells. The mixed cells were seeded on an Ibidi μ-Slide 8 well, and the cells were observed with a Nikon Confocal A1 microscope at 1 hr after mixing. (a) An image with HEK-HER2-iRFP cells (Red). YFP-positive invader cells are shown in green. (b) An image with HEK-iRFP cells (Red). Cell-in-cell structures are indicated by arrows. The scale bars indicate 100 μm.

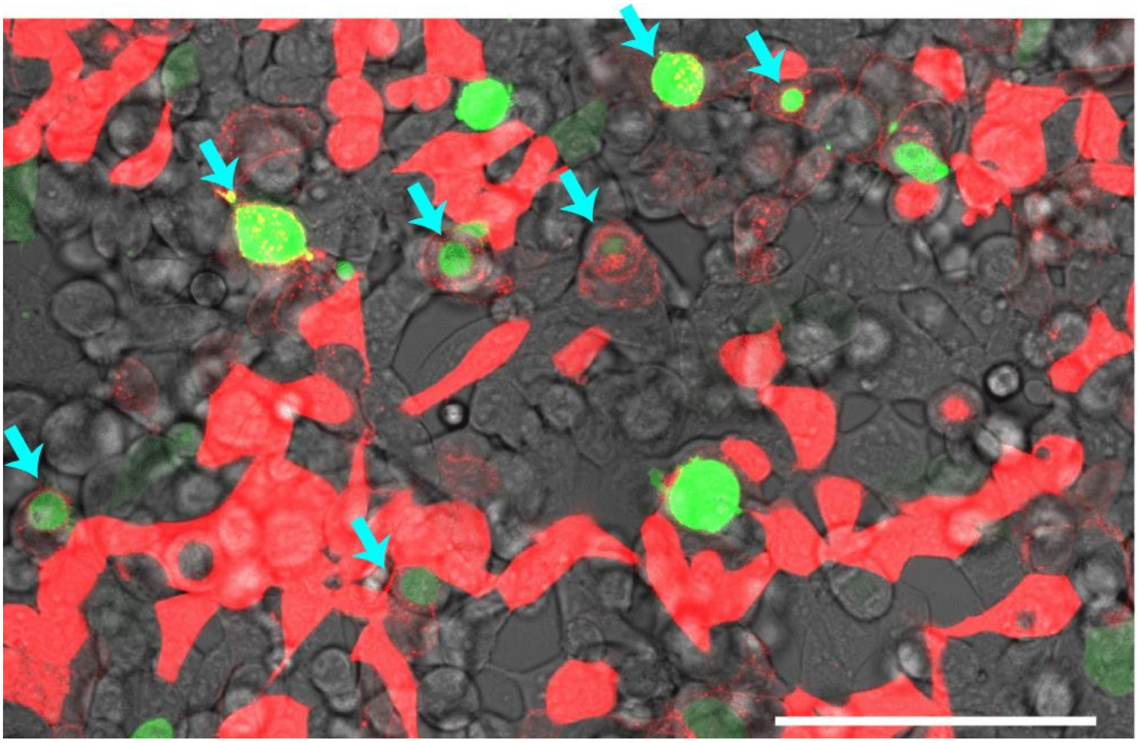

Figure S3. Target-cell-specific invasion in a mixed culture of target cells and non-target cells. The same invader cells as in Fig. S2 were mixed with a mixture of HEK-HER2-iRFP cells and HEK-iRFP cells. The mixed cells were seeded on an Ibidi μ-Slide 8 well, and the cells were observed with a Nikon Confocal A1 microscope at 1 hr after mixing. The cells having a bright red color are HEK-iRFP cells. The cells having a red color at the plasma membrane are HEK-HER2-iRFP cells (HEK-iRFP was considerably brighter than HEK-HER2-iRFP, so fluorescence from HEK-iRFP is saturated to allow visualization of HEK-HER2-iRFP cells). YFP-positive cells (green) are invader cells. Cell-in-cell structures are indicated by arrows. The scale bar indicates 100 μm.

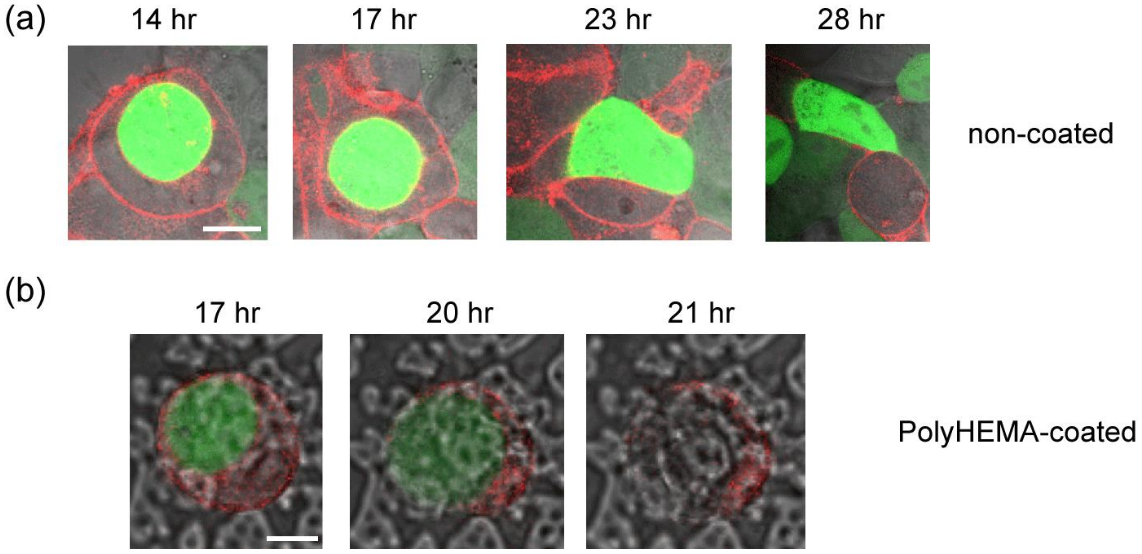

Figure S4: Cell fate observed after cell invasion. HEK-293 cells were transfected with pRK48 (P_{hCMV}-CD43$_{EX}$-RhoA$_{CA}$-pA), pRK47 (P_{hCMV}-ML39-CD28$_{TM}$-RhoA$_{DN}$-pA), and pEYFP-C1 (P_{hCMV}-EYFP-pA) and mixed with HEK-HER2-iRFP cells. The mixed cells were seeded on an Ibidi µ-Slide 8 well (either as purchased (poly-HEMA non-coated) or manually poly-HEMA coated), and the cells were observed with a Nikon Confocal A1 microscope. The time above each image indicates the time after the start of imaging. (a) Escape of an invader cell from a receiver cell. After mixing of the invader and receiver cells, the cells were seeded on an Ibidi µ-Slide 8 well (normal adhesive dish), and time-lapse fluorescence imaging was conducted. Some invader cells escaped from receiver cells, as shown in this figure. (b) Death of an invader cell in a receiver cell. After mixing of the invader and receiver cells, the cells were suspended in warm DMEM containing 0.4% agar, and the suspension were seeded on an Ibidi µ-Slide 8 well (manually coated with poly-HEMA). Then, time-lapse fluorescence imaging was conducted. Most of the invader cells died in the receiver cells. The loss of YFP fluorescence of invader cells could be due to disruption of the plasma membrane and subsequent decrease of intracellular pH (Note that receiver cells try to digest invader cells by lysosomal fusion, and YFP fluorescence decreases at low pH). Error bars indicate 10 µm. See Note S1 for additional comments.

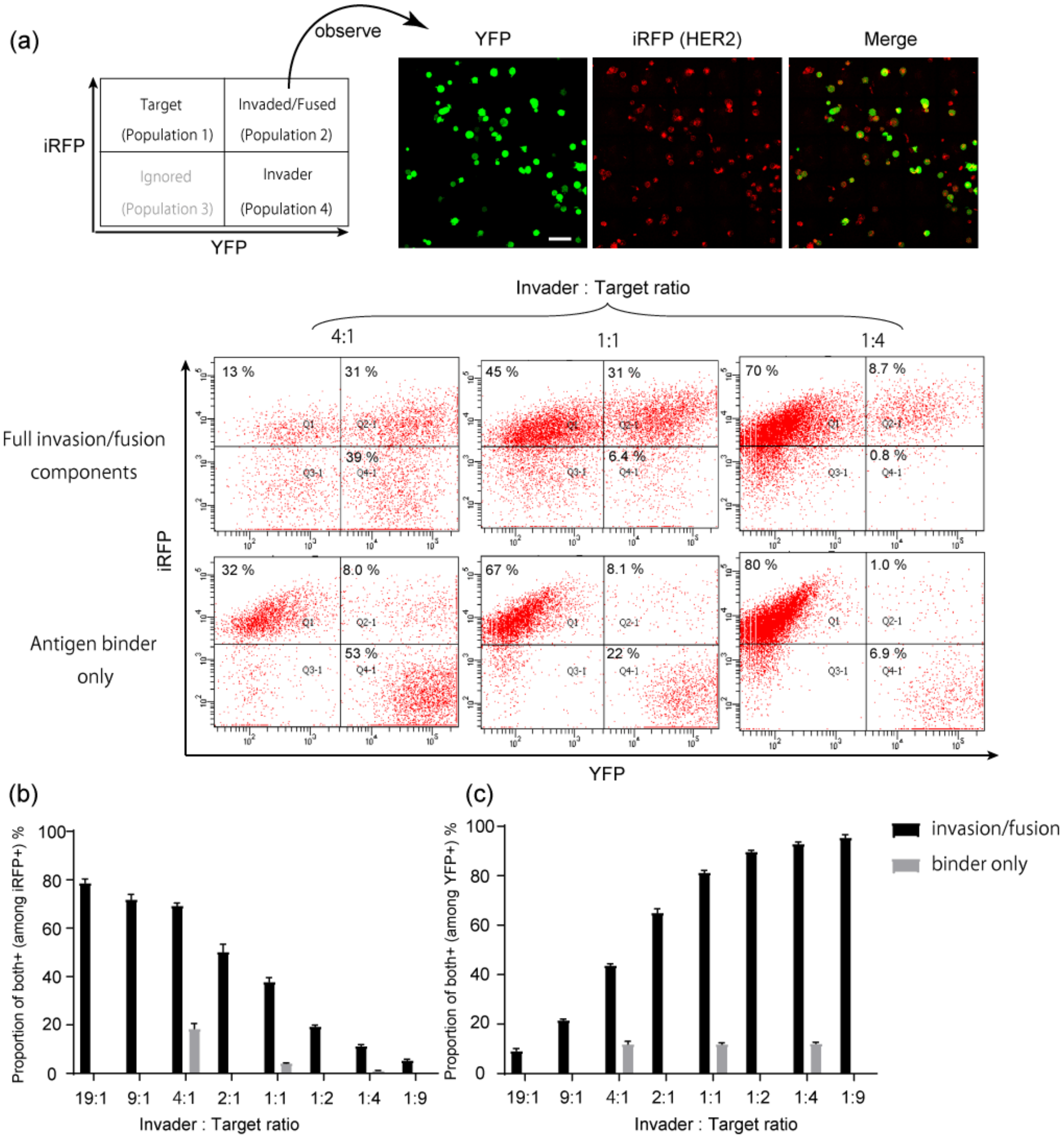

Figure S5: Assessment of the effect of invader : target ratio on invasion/fusion efficacy. This figure shows the details of Fig.2c,d. Note that Fig 2c is the same as Fig S5b, and Fig 2d is the same as Fig S5c. Invader cells were transfected with invasion components (pRK47 and pRK48), VSV-G (pMD2.G) and pEYFP-C1 (for binder-only control, the cells were transfected with the same amount of pRK47 and pEYFP-C1 (using pcDNA3.1(+) as a filler)). At 24 hours after transfection, the invader cells were sorted with a FACS AriaIIIu. The sorted invader cells were mixed with HEK-HER2-iRFP cells at various ratios (19:1~1:9. For the binder-only control, only 4:1, 1:1 and 1:4 ratios were tested). Then, the cells were seeded in 96-well plates (5x10^5 cells/mL in total). After 24 hours, the cells were analyzed with a FACS AriaIIIu. (a) Schematics and representative raw data (as representatives, the results of the FACS analyses with 4:1, 1:1, and 1:4 (invader (or control binder) : target) are shown). iRFP+/YFP- cells (Population 1)

are target cells. iRFP-/YFP+ cells (Population 2) are invader (or control binder) cells. When the cells bearing full invasion/components were used, iRFP+/YFP+ cells (Population 2) were mostly fused cells (when population 4 sorted by FACS was analyzed under a microscope, about 80 % of the population was confirmed to be invaded/fused cells (see upright images); the scale bar indicates 100 μm). iRFP+/YFP+ cells detected while using cells bearing only antigen binder indicate the background of the assay (derived from cell doublets just attaching). (b) The proportion of iRFP+/YFP+ cells among iRFP+ cells (reflecting the proportion of invaded/fused cells among target cells), calculated as Population 2 / (Population 1 + Population 2). Nearly 80 % of target cells became iRFP+/YFP+ when a large excess of invader cells was used. (c) The proportion of iRFP+/YFP+ cells among YFP+ cells (reflecting the proportion of invader cells that had indeed invaded/fused with target cells), calculated as Population 2 / (Population 2 + Population 4). It is shown that nearly all of the invader cells indeed invade target cells, as long as the cells encounter each other. In both (b) and (c), a much smaller proportion of iRFP+/YFP+ cells was detected, confirming that this FACS-based assay worked well. Error bars represent the SEM of three independent experiments (n=3).

Note: The number of invader cells was calculated during the first FACS sorting, and that of target cells was calculated with a Countess Automated Cell Counter (ThermoFisher Scientific). Probably due to the difference of calculation methods (as well as possible cell death after invader cell sorting), the actual cell number of target cells seemed higher (approx. 2-3 times) than that of invader cells even under the 1:1 condition.

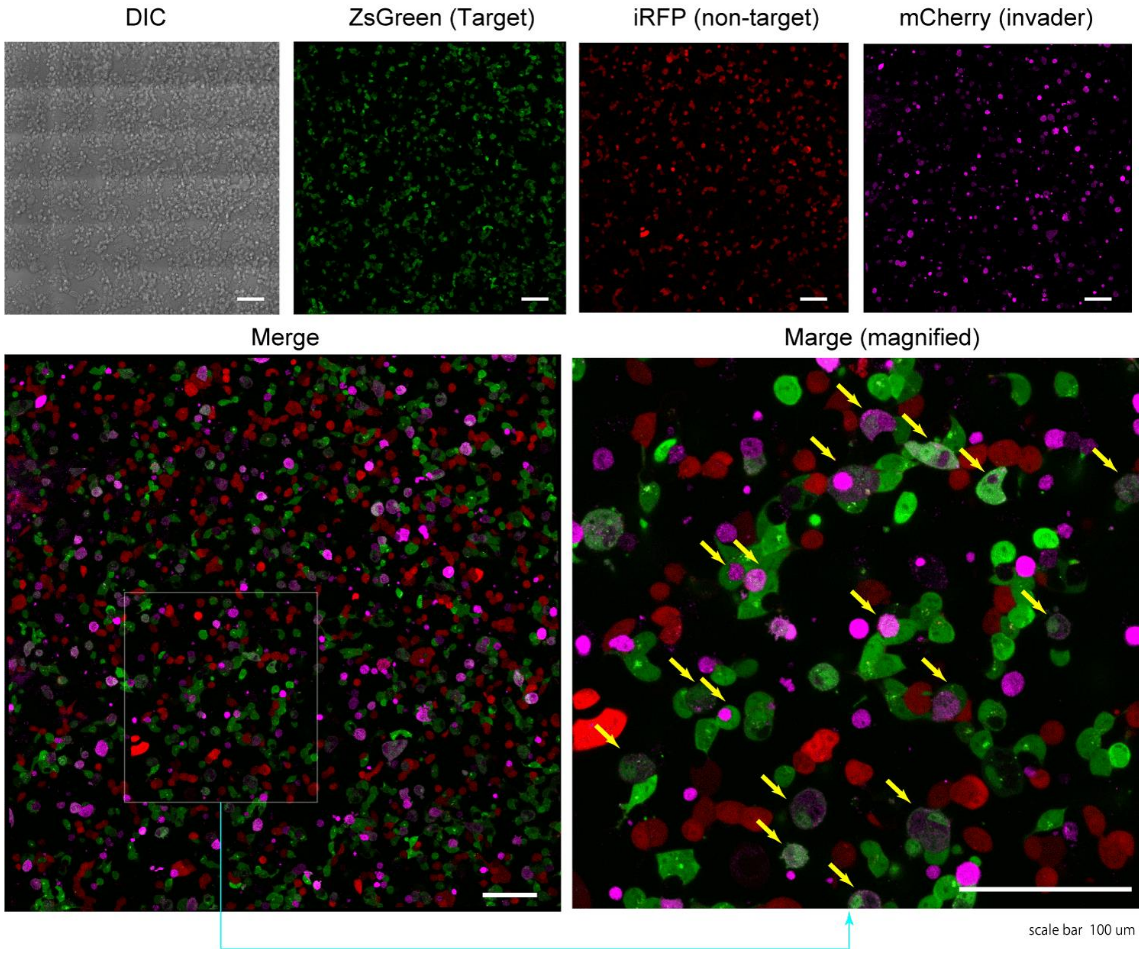

Figure S6: Target-cell-specific invasion/fusion in a mixed culture of target cells and non-target cells. For ease of recognition, HEK-HER2-iRFP-Luc-ZsGreen cells (shown in green) were used as target cells in this experiment. Note that although the HEK-HER2-iRFP-Luc-ZsGreen cells (target) are also iRFP-positive, their iRFP fluorescence is considerably lower than that of HEK-iRFP cells (non-target, shown in red) (as was also the case in Fig.S3), so it could be removed by adjusting the gain of iRFP fluorescence. Invader HEK-293T cells were transfected with pRK48 (P_{hCMV}-$CD43_{EX}$-$RhoA_{CA}$-pA), pRK47 (P_{hCMV}-ML39-$CD28_{TM}$-$RhoA_{DN}$-pA), pMD2.G (P_{hCMV}-VSV-G-pA) and pRK85 (P_{SV40}-mCherry-pA) (shown in magenta), and were mixed with a mixture of target and non-target cells. The mixed cells were seeded on an Ibidi μ-Slide 8 well, and the cells were observed with Leica SP8 microscope at 4 hr after mixing. Invading (invaded) or fused cells are indicated by arrows. Scale bars indicate 100 μm. It can be seen that only target cells are invaded even in the mixed culture. Some of the invaded cells were already fused at this time point.

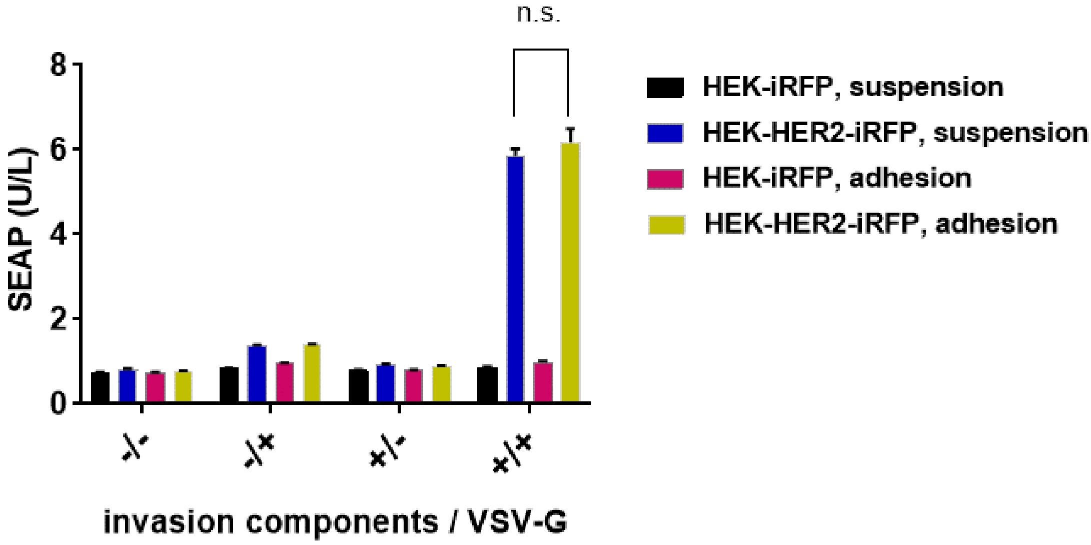

Figure S7. Comparison of the results of tTA delivery-based invasion-fusion assay between a cell-adhesion condition and a cell-suspension condition. As described in the methods section, after mixing the invader HEK-293 cells (± invasion components (pRK47 and pRK48) and VSV-G (pMD2.G), pDB24 was always transfected) and receiver HEK-HER2-iRFP or HEK-iRFP cells (transfected with pMX9), the cell mixture was seeded on either a normal 24-well plate (adhesion) or a poly-HEMA-coated 24-well plate (suspension). SEAP activity in the supernatant was measured at 24 hr after cell seeding. (Values under the "adhesion" condition are the same as in Fig.2c.) As shown here, there was no marked difference. See note S1 for additional comments. Error bars represent SEM of three independent experiments. n.s.: $p>0.05$, two-tailed Student's t-test

a)

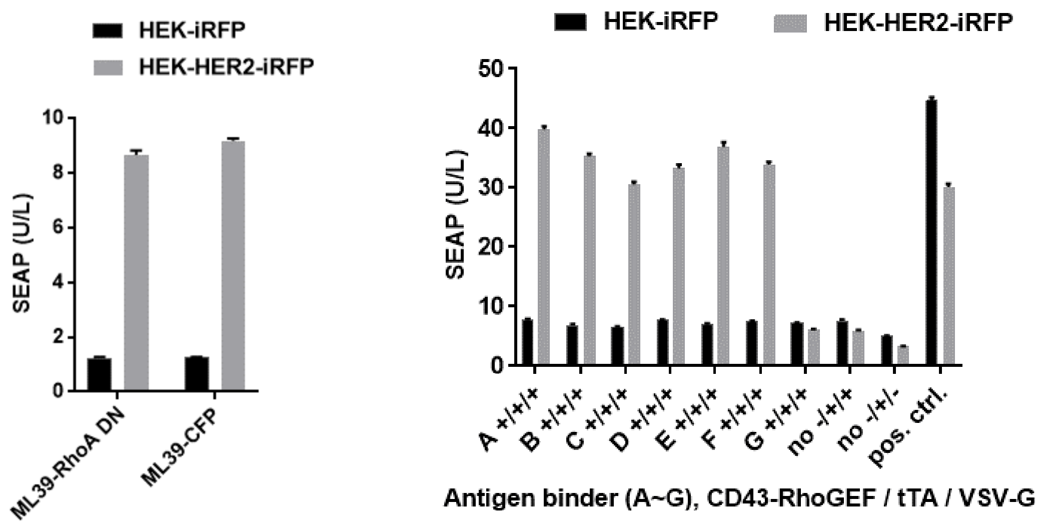

Figure S8: System performance using different antigen binders, transmembrane domains, and an effector. (a) Comparison of ML39-CD28$_{TM}$-RhoA$_{DN}$ and ML39-CD28$_{TM}$-CFP as a binding component. HEK-293 cells were transfected with pRK48 (P$_{hCMV}$-CD43$_{EX}$-RhoA$_{CA}$-pA), pDB24 (P$_{hCMV}$-tTA-pA), pMD2.G (P$_{hCMV}$-VSV-G-pA), and the following plasmid. For ML39-CD28$_{TM}$-RhoA$_{DN}$ condition: pRK47 (P$_{hCMV}$-ML39-CD28$_{TM}$-RhoA$_{DN}$-pA). For ML39-CD28$_{TM}$-CFP condition: pRK34 (P$_{hCMV}$-ML39-CFP-pA). In parallel, HEK-HER2-iRFP and HEK-iRFP cells were transfected with pMX9 (P$_{TET}$-SEAP-pA). The invader cells and the receiver cells were mixed, and seeded on 24-well plates. SEAP activity in the supernatant was measured at 24 hours after seeding. (b) Comparison of different antigen binders and transmembrane domains while using catalytic DH domain of p63RhoGEF as an effector. HEK-293 cells were transfected with the following plasmids (for the + condition; for the – condition, the same amount of pEYFP-C1 was used): pRK253 (P$_{hCMV}$-CD43$_{EX}$-DH(p63RhoGEF)-pA), pDB24 (P$_{hCMV}$-tTA-pA), pMD2.G (P$_{hCMV}$-VSV-G-pA), and a plasmid encoding antigen recognition moiety: A: pRK34 (P$_{hCMV}$-ML39-CD28$_{TM}$-CFP-pA), B: pRK256 (P$_{hCMV}$-ML39-PDGFR$_{TM}$-mCherry-pA), C: pRK200 (P$_{hCMV}$-9_26(anti-HER2 DARPin)-CD28$_{TM}$-CFP-pA), D: pRK259 (P$_{hCMV}$-9_26-PDGFR$_{TM}$-mCherry-pA), E: pRK201 (P$_{hCMV}$-G3(anti-HER2 DARPin)-CD28$_{TM}$-CFP-pA), F: pRK260 (P$_{hCMV}$-G3-PDGFR$_{TM}$-mCherry-pA) G: pRK264 (P$_{hCMV}$-SP6[3](scFv against an irrelevant antigen, synthetic hapten 2,4,6-trinitrophenyl)-CD28$_{TM}$-CFP-pA). In parallel, HEK-HER2-iRFP and HEK-iRFP cells were transfected with pMX9 (P$_{TET}$-SEAP-pA) (for the positive control, pDB24 was co-transfected). The invader cells and the receiver cells were mixed, and seeded on 24-well plates. SEAP activity in the supernatant was measured at 24 hours after seeding the cells. *9_26, G3: Anti-HER2 DARPin[4]. Error bars represent SEM of technical triplicates in a representative experiment.

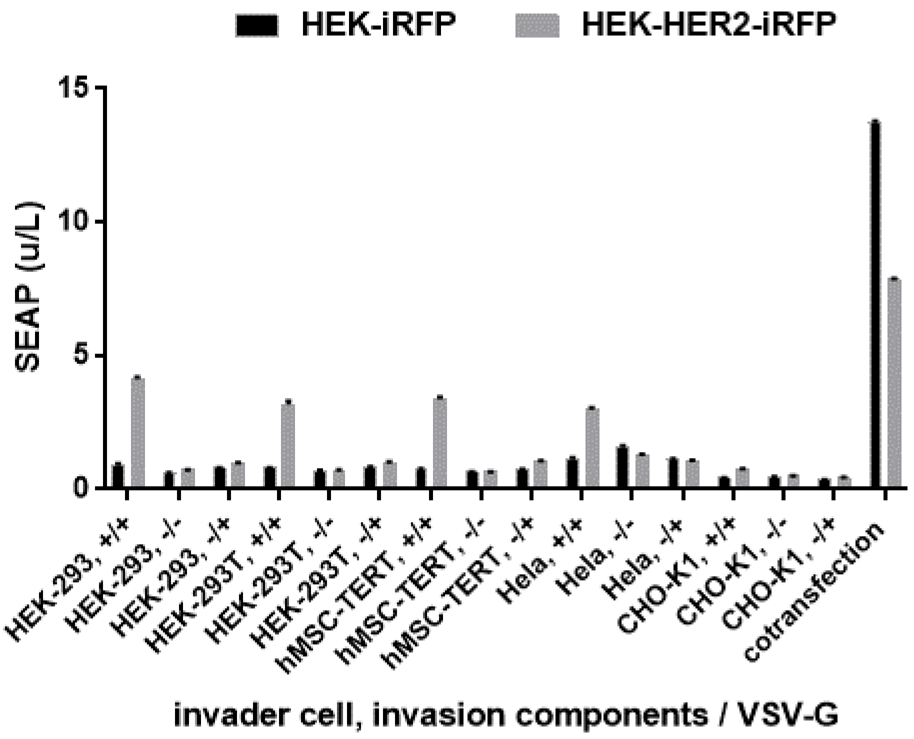

Figure S9: Portability of the cell invasion-fusion system to different cell lines (invader side). Invader cells (HEK-293, HEK-293T, hMSC-TERT, Hela, CHO-K1 cells were transfected with pDB24 (P_{hCMV}-tTA-pA) as well as the following plasmids. Invasion components +: pRK48 (P_{hCMV}-CD43$_{EX}$-RhoA$_{CA}$-pA), and pRK47 (P_{hCMV}-ML39-CD28$_{TM}$-RhoA$_{DN}$-pA). VSV-G +: pMD2.G (P_{hCMV}-VSV-G-pA). For the – condition, pcDNA3.1(+) was used as a filler. In parallel, HEK-HER2-iRFP and HEK-iRFP cells were transfected with pMX9 (P_{TET}-SEAP-pA) (for the co-transfection control, both pDB24 and pMX9 were transfected). The invader cells and the receiver cells were mixed, and seeded on 24-well plates. SEAP activity in the supernatant was measured at 24 hours after seeding the cells. Error bars represent SEM of technical triplicates in a representative experiment.

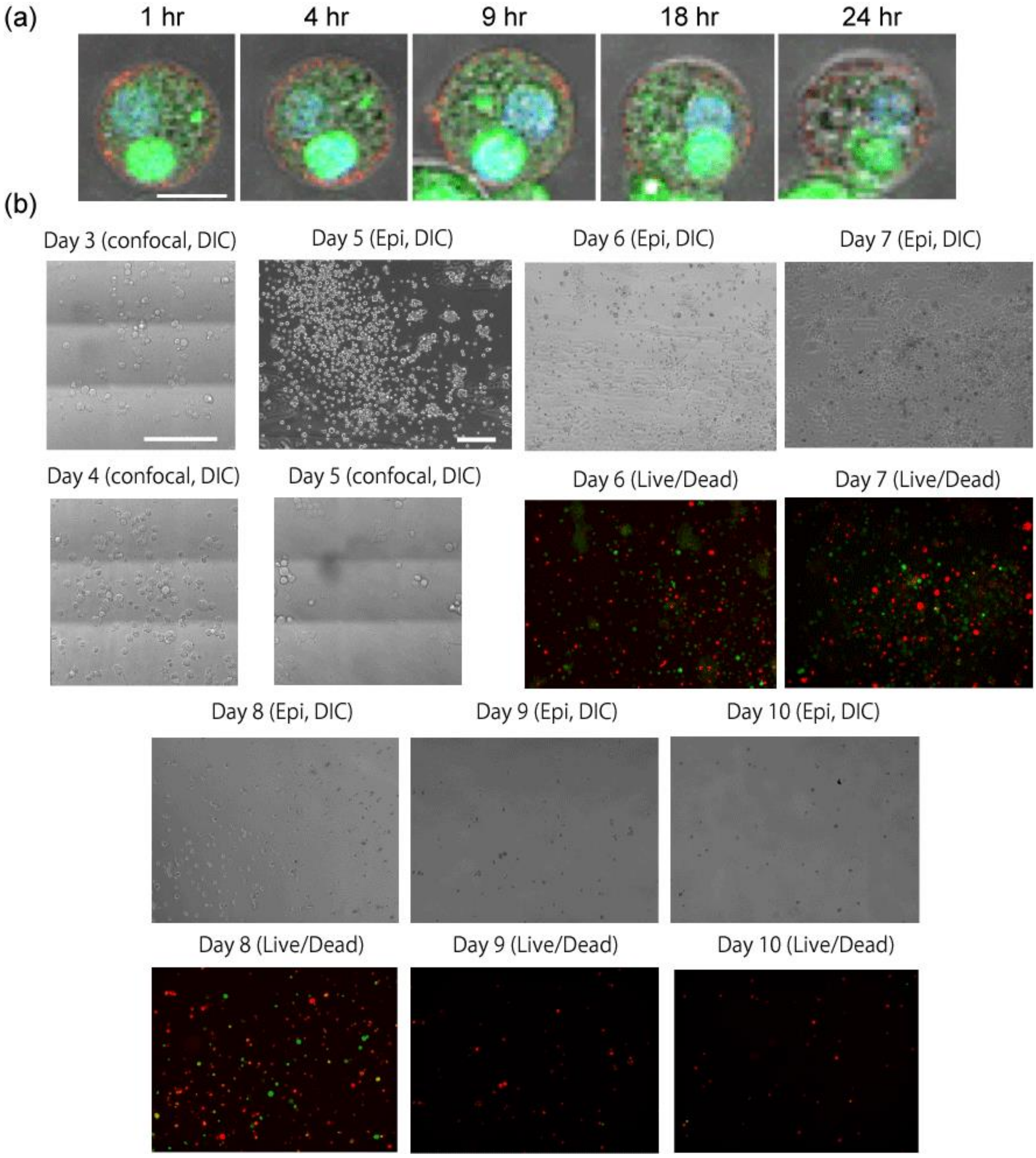

Figure S10. Fate of fused cells. (a) Microscopic observation of nuclei of the fused cells. Invader cells were transfected with pRK48 (P_{hCMV}-CD43$_{EX}$-RhoA$_{CA}$-pA), pRK47 (P_{hCMV}-ML39-CD28$_{TM}$-RhoA$_{DN}$-pA), pDB24 (P_{hCMV}-tTA-pA), pMD2.G ($P_{EF1\alpha}$-tTA-pA), H2b-Citrine (P_{hCMV}-H2b-citrine-pA) and Lyn-YFP (P_{hCMV}-Lyn-YFP-pA). The receiver HEK-HER2-iRFP cells were transfected with pTREtight-dsRed (P_{TET}-dsRed-pA). Also, the nuclei of receiver cells were stained with NlucBlue Live. The invader cells and receiver cells were mixed. After 24 hours, YFP+/iRFP+/DsRed+ cells were sorted by FACS. The sorted cells were seeded on an Ibidi µ-Slide 8 well, and observed under a Nikon Confocal A1 microscope. The red color is due to iRFP (from HEK-HER2-iRFP). The blue nucleus is from a receiver cell (from NlucBlue Live). The bright green nucleus is from an invader cell (from H2b-citrine). The error bar

indicates 10 μm. (b) Invader cells were transfected with pRK48, pRK47, pDB24, pMD2.G and pEYFP-C1. The receiver HEK-HER2-iRFP cells were transfected with pTREtight-dsRed. Then, the invader cells and receiver cells were mixed (Day 0). After 2 days, YFP+/iRFP+/DsRed+ cells were sorted with a FACS (Day 2). The sorted cells were seeded on an Ibidi μ-Slide 8 well, and observed under a Leica SP8 microscope (Confocal) or a Keyence BZ-X700 (Epi) microscope from Day 3 to Day 10 after mixing. At Day 5, most of the fused cells started to float, so the microscope was switched from confocal to epi. From Day 6, the cells were stained with Calcein-AM (2 μM, green, for staining live cells), and EthD-1 (2 μM, red, for staining dead cells) before observation. After Day 7, adherent cells (which were thought to be non-fused cells left as contaminants during cell sorting; the cell sorting efficacy was about 90 %) began to dominate, so the floating cells were re-seeded on a new slide. Note that most of the cells stained red with EthD-1 did not show green fluorescence and most of the cells stained green with Calcein-AM did not show red fluorescence with this gain, so YFP and dsRed transiently transfected first would not have interfered with live-dead staining (Day 6 after cell mixing means 1 week after transfection, so most of the cells were thought to have already lost their fluorescence.) Scale bars indicate 100 μm.

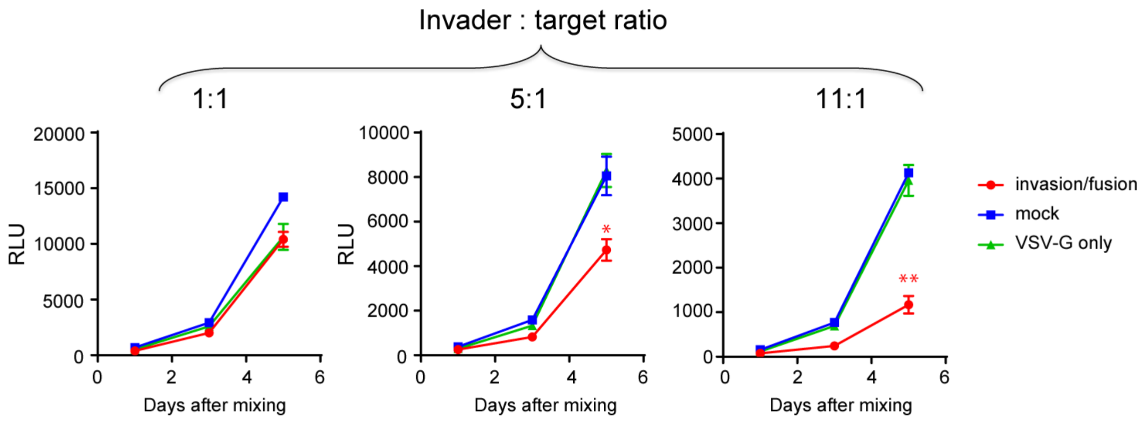

Figure S11. Effect of invader/receiver ratio on the cell killing effect. The same experiment as shown in Fig. 3d was done with different invader cell ratios (1:1, 5:1, 11:1 (Condition "11:1" is the same plot as Fig. 3d)) (total cell number (invader + receiver) was fixed). Error bars represent SEM of three independent experiments (n=3). Statistical analysis was conducted at Day 5. * $p<0.05$, ** $p<0.01$ (against both mock and VSV-G only conditions), two-tailed Student's t-test. Cell killing efficiency appeared to increase as the proportion of invader cells increased. This was thought to be because receiver cells had a greater chance to encounter invader cells. Note that the invader cell population was transiently transfected and included cells that did not take up plasmids; this may account at least in part for the observation that some receiver cells were still growing even at the ratio of 11:1.

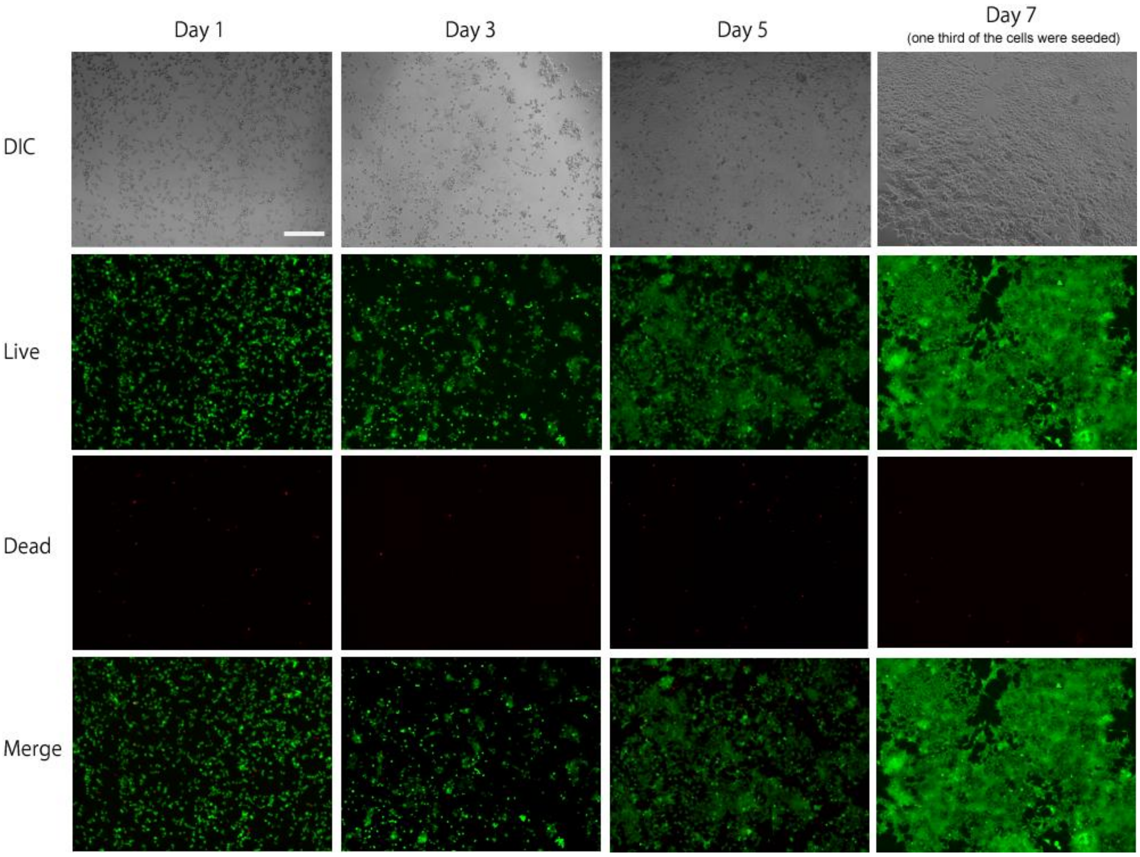

Figure S12. Assessment of the viability of invader cells. HEK-293T cells were transfected with pRK48 (P_{hCMV}-CD43$_{EX}$-RhoA$_{CA}$-pA), pRK47 (P_{hCMV}-ML39-CD28$_{TM}$-RhoA$_{DN}$-pA), and pRK85 (P_{hCMV}-iRFP-pA). Then cells were sorted with a FACS (based on iRFP fluorescence) at 24 hours after transfection. Then, the cells were seeded on an Ibidi μ-Slide 8 well ($2.5 x 10^5$ cells/mL for observation at Days 1-5, $0.8 x 10^5$ cells/mL for observation at Day 7, 250 μL/well). At 1, 3, 5 or 7 days after seeding, the cells were stained with Calcein-AM (2 μM, green, for live cells), and EthD-1 (2 μM, red, for dead cells), and the fluorescence was observed with a Keyence BZ-X700 microscope. The error bar indicates 100 μm.

Note S1: Note concerning the cell-suspension effect.

Entotic cell engulfment is induced by matrix detachment[5], so the surface condition of cell culture dish was expected to be an important factor in our experiments. In our protocols, we first mixed the invader cells and receiver cells in a 1.5 mL tube. The mixture was incubated for 30 min, and then seeded on a plate or slide (either cell-adhesive condition (normal dish) or cell-suspension condition (dish coated with poly-HEMA). Since the invasion process was relatively fast, we could see cell-in-cell structures with a normal adherent dish. Cell fusion after invasion was also a relatively fast process, and there was no significant difference in the result of tTA transfer-based reporter gene assay between cell-adhesive and cell-suspension conditions (Fig.S7). So, we used a normal cell-adhesive dish for most of the experiments unless otherwise specified. There was a difference in the major cell fate after cell invasion (without VSV-G expression, Fig.S4a,b). In the cell-adhesive condition, escape of the engulfed cells from the receiver cells was more frequently observed, as compared with the suspension condition (in the suspension condition, cell death of the engulfed cell seemed to dominate). So, we show results using both the adhesion condition and the suspension condition in Fig.S4 and S7.

Table S1: Description of the plasmids used in this study.

Plasmid	Description and Cloning Strategy	Reference/Source
**pRK14**	Constitutive $CD43_{EX}$-YFP expression vector (P_{hCMV}-$CD43_{EX}$-YFP-pA_{SV40}).	Kojima et al[2]
**pRK21**	Constitutive HER2-iRFP670 expression vector (P_{hCMV}-HER2-iRFP-pA_{bGH}).	Kojima et al[2]
**pRK22**	Constitutive iRFP670 expression vector (P_{hCMV}-iRFP-pA_{bGH}).	Kojima et al[2]
**pRK34**	Constitutive Igκ leader signal-ML39-CD28 transmembrane domain –CFP expression vector (P_{hCMV}-ML39-$CD28_{TM}$-CFP-pA_{bGH}). DNA fragments encoding Igκ leader signal-ML39, $CD28_{TM}$, and CFP were PCR amplified as follows. Igκ leader signal-ML39: The fragment was amplified by oRK41 and oRK9 by using addgene #10794, a gift from Judy Lieberman[6] as a template. This fragment was then digested with *Nhe*I/*Hin*dIII. $CD28_{TM}$: The fragment (additionally having cMyc) was amplified by using oRK16 and oRK17 as primers and pAT04 (unpublished, a gift from Aizhan Tastan. This construct encodes a chimeric antigen receptor bearing scFv SP6 provided by Steven A. Rosenberg[3]) as a template. This fragment was then digested with *Hin*dIII/*Eco*RI. CFP: The fragment (having GGSGG linker in the N-terminal side of CFP) was amplified by oRK22 and oRK23 by using pECFP-C1 (Clonetech) as a template. The fragment was then digested with *Eco*RI/*Not*I. These 3 fragments were stepwisely inserted into corresponding sites of pcDNA3.1(+).	This work
**pRK47**	Constitutive Igκ leader signal-ML39-CD28 transmembrane domain –dominant negative RhoA (RhoA T19N, devoid of C-termial CAAX domain) expression vector (P_{hCMV}-ML39-$CD28_{TM}$-RhoA DN-pA_{bGH}). The fragment coding RhoA T19N devoid of CAAX domain (+additional HA tag in the C-termunus) was PCR amplified by using oRK63 and oRK64 as primers, and addgene #12967 (a gift from Gary Bokoch[7]) as a template. The amplified fragment was digested with *Eco*RI/*Apa*I, and was inserted into pRK34 digested with *Eco*RI/*Apa*I.	This work
**pRK48**	Constitutive $CD43_{EX}$-constitutively active RhoA (RhoA Q63L, devoid of CAAX domain) expression vector (P_{hCMV}-$CD43_{EX}$-RhoA	This work

	CA-pA$_{SV40}$). The fragment coding RhoA Q63L devoid of C-terminal CAAX domain (+additional FLAG tag) was PCR amplified by using oRK65 and oRK66 as primers, and addgene #12968 (a gift from Gary Bokoch[7]) as a template. The amplified fragment was digested with *Age*I/*Apa*I, and was inserted into pRK14 digested with *Age*I/*Apa*I.	
**pRK64**	Constitutive full length RhoA T19N (dominant negative) expression vector (P$_{hCMV}$-RhoA DN-pA$_{bGH}$). RhoA T19N was PCR amplified by using oRK86 and oRK43 as primers, and addgene #12967 as a template. The amplified fragment was digested with *Hin*dIII/*Apa*I, and was inserted into corresponding site of pcDNA3.1(+).	This work
**pRK65**	Constitutive full length RhoA Q63L (constitutively active) expression vector (P$_{hCMV}$-RhoA CA-pA$_{bGH}$). RhoA Q63L was PCR amplified by using oRK86 and oRK43 as primers, and addgene #12968 as a template. The amplified fragment was digested with *Hin*dIII/*Apa*I, and was inserted into corresponding site of pcDNA3.1(+).	This work
**pRK66**	Constitutive Igκ leader signal-ML39-CD28 transmembrane domain –constitutively active RhoA (RhoA Q63L, devoid of C-termial CAAX domain) expression vector (P$_{hCMV}$-ML39-CD28$_{TM}$-RhoA CA-pA$_{bGH}$). The fragment coding RhoA Q63L devoid of CAAX domain (+additional HA tag in the C-termunus) was PCR amplified by using oRK63 and oRK64 as primers, and pRK48 as a template. The amplified fragment was digested with *Eco*RI/*Apa*I, and was inserted into pRK34 digested with *Eco*RI/*Apa*I.	This work
**pRK67**	Constitutive CD43$_{EX}$-dominant negative RhoA (RhoA T19N, devoid of CAAX domain) expression vector (P$_{hCMV}$-CD43$_{EX}$-RhoA CA-pA$_{SV40}$). The fragment coding RhoA T19N devoid of C-terminal CAAX domain (+additional FLAG tag) was PCR amplified by using oRK65 and oRK66 as primers, and addgene pRK47 as a template. The amplified fragment was digested with *Age*I/*Apa*I, and was inserted into pRK14 digested with *Age*I/*Apa*I.	This work
**pRK85**	A plasmid vector for constitutive expression (driven by P$_{SV40}$) of mCherry. The fragment coding mCherry was PCR amplified by using oRK110 and oRK111 from a mCherry expressing plasmid. The amplified fragment was digested with XmaI/BstBI and was inserted into pcDNA3.1(+) digested with *Xma*I/*BstB*I.	This work
**pRK200**	Constitutive Igκ leader signal-9_26[4] (anti-HER2 DARPin)-CD28 transmembrane domain–CFP expression vector (P$_{hCMV}$-9_26-CD28$_{TM}$-CFP-pA$_{bGH}$). The fragment coding 9_26 was PCR amplified by using oRK197 and oRK198 as primers, and 9_26_in_pQE30_2xstop_(corr31) (a gift from Pluckthun lab, ETH Zurich) as a template. The amplified fragment was digested with *Nhe*I/*Hin*dIII. This fragment was and was inserted into pRK34 digested with *Nhe*I/*Hin*dIII.	This work
**pRK201**	Constitutive Igκ leader signal-G3[8] (anti-HER2 DARPin)-CD28 transmembrane domain–CFP expression vector (P$_{hCMV}$-G3-CD28$_{TM}$-CFP-pA$_{bGH}$). The fragment coding G3 was PCR amplified by using oRK197 and oRK198 as primers, and pDST072_corr (a gift from Pluckthun lab, ETH Zurich) as a template. The amplified fragment was digested with *Nhe*I/*Hin*dIII. This fragment was and was inserted into pRK34 digested with *Nhe*I/*Hin*dIII.	This work
**pRK253**	Constitutive CD43$_{EX}$-DH domain of p63RhoGEF expression vector (P$_{hCMV}$-CD43$_{EX}$-DH(p63RhoGEF)-pA$_{SV40}$). The fragment coding DH domain of p63RhoGEF was PCR amplified by using oRK258 and oRK259 as primers, and DNASU (HsCD00441667) as a template. The amplified fragment was digested with *Age*I/*Apa*I, and was inserted into pRK48 digested with *Age*I/*Apa*I.	This work
**pRK256**	Constitutive Igκ leader signal-ML39-PDGFR transmembrane domain–mCherry expression vector (P$_{hCMV}$-ML39-PDGFR$_{TM}$-mCherry-pA$_{bGH}$). The fragment coding Igκ-ML39 was cut out from pRK34 by *Sac*I/*Hind*III, and was inserted into pLeo404 (unpublished, a gift from Leo Scheller, coding transmembrane domain of PDGFR and C-terminal mCherry) digested with *Sac*I/*Hin*dIII.	This work

**pRK259**	Constitutive Igκ leader signal-9_26-PDGFR transmembrane domain–mCherry expression vector (P_{hCMV}-9_26-$PDGFR_{TM}$-mCherry-pA_{bGH}). The fragment coding Igκ-9_26 was cut out from pRK200 by *Sac*I/*Hind*III, and was inserted into pLeo404 digested with *Sac*I/*Hin*dIII.	This work
**pRK260**	Constitutive Igκ leader signal-G3-PDGFR transmembrane domain–mCherry expression vector (P_{hCMV}-G3-$PDGFR_{TM}$-mCherry-pA_{bGH}). The fragment coding Igκ–G3 was cut out from pRK201 by *Sac*I/*Hind*III, and was inserted into pLeo404 digested with *Sac*I/*Hin*dIII.	This work
**pRK264**	Constitutive SP6-PDGFR transmembrane domain–mCherry expression vector (P_{hCMV}-SP6-$PDGFR_{TM}$-mCherry-pA_{bGH}). SP6 (including secretion signal sequence) was PCR amplified by using oRK174 and oRK175 as primers and pAT04 as a template. This fragment was digested with *Nhe*I/*Hin*dIII. Also, a fragment coding $CD28_{TM}$-RhoA DN was cut out from pRK47 with *Hin*dIII/*Apa*I. These 2 fragments were inserted in pcDNA3.1(+) digested with *Nhe*I/*Apa*I, yielding pRK143 (P_{hCMV}-SP6-$CD28_{TM}$-RhoA DN-pA_{bGH}). A fragment coding SP6 was cut out from pRK143 by *Sac*I/*Hin*dIII, and was inserted into pLeo404 digested with *Sac*I/*Hin*dIII.	This work
**pDB24**	Constitutive tTA expression vector (P_{hCMV}-tTA-pA_{SV40}). tTA was PCR amplified by using oDB23 and oDB24 as primers and pSAM200[9] as a template. This fragment was digested with *Nhe*I/*Not*I, and was inserted into pEGFP-N1 (Clontech) digested with *Nhe*I/*Not*I.	This work
**pMX9**	tTA responsive SEAP expression vector (P_{TET}-SEAP-pA).	Folcher et al[10]
**pcDNA3.1(+)**	Empty vector bearing hCMV promoter and multicloning site (MCS). (P_{hCMV}-MCS-pA_{bGH}).	Invitrogen
**pEYFP-C1**	Constitutive EYFP expression vector. (P_{hCMV}-EYFP-pA_{SV40}).	Clontech
**pMD2.G**	Constitutive VSV-G expression vector (P_{hCMV}-(β-globin intron)-VSV-G-$pA_{\beta\text{-globin}}$. (a gift from Didier Trono)	Addgene #12259
**pTREtight-dsRed**	tTA responsive dsRed expression vector (P_{TET}-dsRed-pA_{SV40}) (Offcial name: pTREtight-BI-dsRed-Express)	Clontech
**H2b-citrine**	Constitutive H2b-Citrine (nucleus-localizing citrine) expression vector	A gift from Marius Muller
**Lyn-YFP**	Constitutive lyn-YFP (plasmamembrane-localizing EYFP) expression vector	A gift from Takanari Inoue

Table S2: Table of the oligonucleotides used for cloning.

**Oligo name**	Sequence
**oRK6**	accacggcagccagctctctggagac
**oRK7**	ctgaACCGGTcctccagcgccaccagtccgccgcttctgccg
**oRK9**	ATCGaagcttACCTAGGACGGTCAGCTTGGTTCCTC
**oRK16**	CAGTaagcttGAACAAAAACTCATCTCAGAAGAGGATCTGGTGAAAGGGAAACACCTTTGTCCAAGTCCC
**oRK17**	GTGCgaattcCTTACTCCTCACCCAGAAAATAATAAAGGCCACTGT
**oRK22**	atgaGAATTCggtggctccggaggaATGGTGAGCAAGGGCGAGGAGCTG
**oRK23**	ATTAgcggccgcCTTGTACAGCTCGTCCATGCCGAGAGT
**oRK41**	taatGCTAGCgccaccATGGAGACAGACACACTCCTGCTATGGGTACTGCTGCTCTGGGTTCCAGGTTCCACTGGTGACATGGCCCAGGTGCAGCTGGT
**oRK43**	ATGCgggcccTCACAAGACAAGGCAACCAGATTTTTTCTTCCC
**oRK63**	attaGAATTCggtagtgctggtggtATGGCTGCCATCCGG
**oRK64**	taacGGGCCCttaAGCGTAATCTGGAACATCGTATGGGTAACCAGATTTTTTCTTCCCACGTCTAGCTTGCAG
**oRK65**	attcACCGGTCGCtACaATGGCTGCCATCCGGAAGAAACTGG
**oRK66**	attcGGGCCCttaCTTGTCGTCATCGTCTTTGTAGTCACCAGATTTTTTCTTCCCACGTCTAGCTTGCAG
**oRK86**	attcAAGCTTgccaccATGGCTGCCATCCGGAAGAAACTGG
**oRK110**	ATTAcccgggactagtGCCACCatggtgagcaagggcgaggagg
**oRK111**	attgTTCGAAttacttgtacagctcgtccatgccgcc
**oRK174**	TAATgctagcgccaccATGACCCAGTCTCCAAAATTCATGTCC
**oRK175**	TAGAaagcttCGTAGTTCCTTGGCCCCAGTAAGCAAG

**oRK197**	attgGCTAGCTTAATTAAgccaccATGGAGACAGACACACTCCTGCTATGGGTACTGCTGCTCTGGGTTCCAGGTTCCACTGGTGACAGAGGATCGCATCACCATCACCATCACG
**oRK198**	ATTAgaattcATTAAGCTTTTGCAGGATTTCAGCCAGGTCC
**oRK258**	attgACCGGTgggaggctcaggtggcAAGAAGGCTCTGGAAAGGAGTATGTATGTCCTGAG
**oRK259**	taatGGGCCCttaGCCCTCAAATCCCCGCAATCTCCC
**oDB23**	ATATgctagcGCCACCATGTCAAGATTAGATAAAAGTA
**oDB24**	ATATgcggccgcTTAACCACCGTACTCGTCAATTCCAA

Supporting references

[1] J. L. Simonsen, C. Rosada, N. Serakinci, J. Justesen, K. Stenderup, S. I. Rattan, T. G. Jensen, M. Kassem, *Nat. Biotechnol.* **2002**, 20, 592-596.

[2] R. Kojima, L. Scheller, M. Fussenegger, *Nat. Chem. Biol.* **2018**, 14, 42-49.

[3] D. Chinnasamy, Z. Y. Yu, M. R. Theoret, Y. B. Zhao, R. K. Shrimali, R. A. Morgan, S. A. Feldman, N. P. Restifo, S. A. Rosenberg, *J. Clin. Invest.* **2010**, 120, 3953-3968.

[4] D. Steiner, P. Forrer, A. Pluckthun, *J. Mol. Biol.* **2008**, 382, 1211-1227.

[5] M. Overholtzer, A. A. Mailleux, G. Mouneimne, G. Normand, S. J. Schnitt, R. W. King, E. S. Cibas, J. S. Brugge, *Cell* 2007, 131, 966-979.

[6] E. Song, P. Zhu, S. K. Lee, D. Chowdhury, S. Kussman, D. M. Dykxhoorn, Y. Feng, D. Palliser, D. B. Weiner, P. Shankar, W. A. Marasco, J. Lieberman, *Nat. Biotechnol.* **2005**, 23, 709-717.

[7] M. C. Subauste, M. Von Herrath, V. Benard, C. E. Chamberlain, T. H. Chuang, K. Chu, G. M. Bokoch, K. M. Hahn, *J. Biol. Chem.* **2000**, 275, 9725-9733.

[8] C. Zahnd, E. Wyler, J. M. Schwenk, D. Steiner, M. C. Lawrence, N. M. McKern, F. Pecorari, C. W. Ward, T. O. Joos, A. Pluckthun, *J. Mol. Biol.* **2007**, 369, 1015-1028.

[9] M. Fussenegger, S. Moser, X. Mazur, J. E. Bailey, *Biotechnol. Prog.* **1997**, 13, 733-740.

[10] M. Folcher, M. Xie, A. Spinnler, M. Fussenegger, *Nucl. Acids Res.* **2013**, 41, e134.
